# An integrated functional and clinical genomics approach reveals genes driving aggressive metastatic prostate cancer

**DOI:** 10.1038/s41467-021-24919-7

**Published:** 2021-07-29

**Authors:** Rajdeep Das, Martin Sjöström, Raunak Shrestha, Christopher Yogodzinski, Emily A. Egusa, Lisa N. Chesner, William S. Chen, Jonathan Chou, Donna K. Dang, Jason T. Swinderman, Alex Ge, Junjie T. Hua, Shaheen Kabir, David A. Quigley, Eric J. Small, Alan Ashworth, Felix Y. Feng, Luke A. Gilbert

**Affiliations:** 1 Department of Radiation Oncology, University of California, San Francisco, San Francisco, CA USA; 2 Helen Diller Family Comprehensive Cancer Center, University of California, San Francisco, San Francisco, CA USA; 3 Department of Urology, University of California, San Francisco, San Francisco, CA USA; 4 Division of Hematology and Oncology, Department of Medicine, University of California, San Francisco, San Francisco, CA USA; 5 Department of Epidemiology and Biostatistics, University of California, San Francisco, San Francisco, CA USA; 6 Department of Cellular & Molecular Pharmacology, University of California, San Francisco, San Francisco, CA USA

**Keywords:** Cancer genomics, Prostate cancer

## Abstract

Genomic sequencing of thousands of tumors has revealed many genes associated with specific types of cancer. Similarly, large scale CRISPR functional genomics efforts have mapped genes required for cancer cell proliferation or survival in hundreds of cell lines. Despite this, for specific disease subtypes, such as metastatic prostate cancer, there are likely a number of undiscovered tumor specific driver genes that may represent potential drug targets. To identify such genetic dependencies, we performed genome-scale CRISPRi screens in metastatic prostate cancer models. We then created a pipeline in which we integrated pan-cancer functional genomics data with our metastatic prostate cancer functional and clinical genomics data to identify genes that can drive aggressive prostate cancer phenotypes. Our integrative analysis of these data reveals known prostate cancer specific driver genes, such as *AR* and *HOXB13*, as well as a number of top hits that are poorly characterized. In this study we highlight the strength of an integrated clinical and functional genomics pipeline and focus on two top hit genes, *KIF4A* and *WDR62*. We demonstrate that both *KIF4A* and *WDR62* drive aggressive prostate cancer phenotypes in vitro and in vivo in multiple models, irrespective of AR-status, and are also associated with poor patient outcome.

## Abstract (web summary)

It is hypothesized that there are a number of tumor specific driver genes for metastatic prostate cancer. Here, the authors perform genome-wide CRISPRi screens and integrate these data with metastatic prostate cancer functional and clinical genomics data to show that KIF4A and WDR62 drive aggressive prostate cancer phenotypes.

## Introduction

Prostate cancer is a common cancer and the second leading cause of cancer-related deaths among men in the United States^1^. Androgens are a key driver of prostate cancer cell proliferation, and androgen deprivation therapy (ADT) is the mainstay of treatment for men with metastatic prostate cancer^2,3^. While ADT is initially effective, metastatic prostate cancer patients on ADT will ultimately develop resistance and inevitably progress to lethal metastatic castration-resistant prostate cancer (mCRPC)^4^. There is a critical unmet need to identify new molecular therapeutic targets for patients with mCRPC. We sought to address this issue using both functional genomics and clinical genomics strategies to identify driver genes that are associated with disease progression.

Advances in DNA sequencing have enabled comprehensive genomic analysis of metastatic tumors and have identified adaptive changes in the underlying molecular signaling pathways associated with mCRPC^5–7^. Similarly, the advent of loss-of-function CRISPR functional genomics platforms has systematically revealed which genes are required for cancer cell proliferation and survival^8,9^. Although both of these approaches have generated substantial amounts of data, it remains unclear how best to utilize these strategies to identify driver genes that are specific to the context of a particular disease, such as mCRPC. In principle, the combination of clinical and functional genomics data enables one to distinguish universally essential genes from genes that drive specific cancers that are associated with poor prognosis. We therefore hypothesized that an integrated approach could nominate innovative therapeutic targets for patients with aggressive prostate cancer.

We first performed genome-scale CRISPR-interference (CRISPRi) screens in metastatic prostate cancer models to identify genes required for cellular proliferation or survival. We then created an analytic pipeline that integrated mCRPC functional and clinical genomics data with pan-cancer CRISPR functional genomics data, and in doing so identified a number of previously undescribed prostate cancer-specific driver genes. We demonstrated that two of these genes, *KIF4A* (Kinesin Family Member 4A) and *WDR62* (WD Repeat Domain 62), promote aggressive prostate cancer phenotypes in vitro and in vivo. These experiments, combined with clinical data on these genes, serve to nominate *KIF4A* and *WDR62* as prostate cancer-specific driver genes.

## Results

### Genome-scale CRISPRi screens identify prostate cancer-specific driver genes

**Fig. 1:**
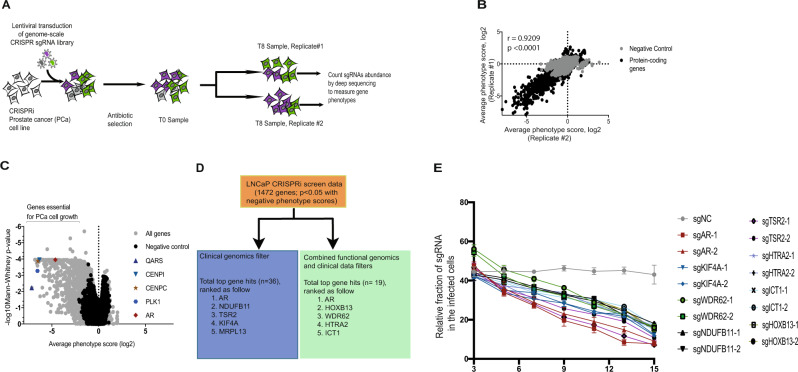
Genome-scale CRISPRi screen to identify prostate cancer-specific driver genes. **A** Schematic of growth-based CRISPRi screens; **B** Correlation plot showing concordance between replicates of the CRISPRi screen in LNCaP cells (LNCaPi) at gene level; **C** Volcano plot showing Mann–Whitney statistical significance and average phenotype score of all genes and negative controls; **D** Filtering strategies implemented to identify genes that are specific to prostate cancer biology in the context of metastasis; **E** Competitive growth-based assay to validate top eight hits (*n* = 3 as biological replicates; Mean ± SEM).

We performed genome-scale screens using our previously described CRISPRi functional genomics platform^10,11^ in two mCRPC cell lines, LNCaP and C42B, to identify genes that are required for prostate cancer cell proliferation or survival (Fig. 1A). To begin, we generated multiple malignant and immortalized benign prostate CRISPRi cell-line models that stably express dCas9-BFP-KRAB fusion proteins (Supplementary Fig. 1). LNCaP and C42B cell lines are excellent preclinical in vitro models to study mCRPC^12^. These cell models represent androgen-sensitive (LNCaP) and androgen-insensitive aggressive (C42B) mCRPC. Genome-scale pooled genetic screens were performed by transducing LNCaP or C42B cells stably expressing dCas9-KRAB (hereafter denoted as LNCaPi and C42Bi) with a human genome-scale CRISPRi library^10,11^ (Fig. 1A and Supplementary Data 1). Samples were collected at time zero (T0) and after 8 population doublings (T8) (Fig. 1A). Each screen was performed in duplicate. We then used next-generation sequencing to quantify the abundance of the sgRNAs in each population of cells. Results obtained from the replicate screens were highly correlated (Fig. 1B and Supplementary Fig. 2A, B). The LNCaP and C42B CRISPRi screens had many shared and selective genetic dependencies (Supplementary Fig. 2C). We were primarily interested in androgen-sensitive mCRPC and so prioritized the LNCaPi screen data. Analysis of our LNCaPi screen data revealed 1472 genes that are required for cell proliferation or survival (Fig. 1C and Supplementary Data 2). Given that the vast majority of these genes were expected to be generally required for cell proliferation, rather than being specifically essential in prostate cancer, we next developed a bioinformatic strategy to identify prostate cancer-specific driver genes.

In order to identify driver genes that are specific to aggressive prostate cancer, we applied a clinical genomics filter designed to prioritize CRISPRi hits with evidence of genomic amplification and/or increased gene expression. This analysis strategy revealed the Androgen Receptor (AR) as the top prostate cancer-specific gene hit, but the next four top-ranked hits were genes not previously associated with prostate cancer: *KIF4A, MRPL13, NDUFB11,* and *TSR2* (top 5) (Fig. 1D and Supplementary Data 3). Examination of pan-cancer functional genomics data^13,14^ for essentiality phenotypes for the top 5 hits revealed that AR is selectively essential for prostate cancer models as expected (Supplementary Fig. 3A). In contrast, top hits such as *TSR2* are essential for the proliferation or survival of nearly all cell types and thus are almost certainly not prostate cancer-specific driver genes (Supplementary Fig. 3B). Other top hits such as *KIF4A, MRPL13,* and *NDUFB11* are essential for a number of cell types but not for others (Supplementary Fig. 3C–E). These results demonstrated that solely using clinical data to filter loss-of-function CRISPR functional genomics data can identify known driver genes but also generates a significant rate of false-positive hits as exemplified by TSR2 in this analysis.

Given these results, we next tested whether a combined clinical and functional genomics filtering strategy would more robustly reveal prostate cancer-specific driver genes in our LNCaPi screen data. We first filtered our list of 1472 LNCaP essential genes against a CRISPR pan-cancer functional genomics datasets (pooled in vitro CRISPR knockout library essentiality screens (PICKLES) and DepMap datasets)^13,15^. We then filtered the remaining hits against two published non-prostate cancer CRISPRi screens^16^ to remove CRISPR DepMap false negatives in the prostate cancer CRISPRi screen data. Lastly, we prioritized genes associated with increased expression in metastatic prostate cancer samples (Fig. 1D and Supplementary Data 4). This integrative functional genomics and clinical genomics filtering strategy revealed known prostate cancer-specific driver genes, *AR* and *HOXB13*, as the top two hits^5,7,17,18^. Additional top hits, such as *WDR62*, are uncharacterized in prostate cancer (Fig. 1D). These results demonstrate that an integrated functional genomics and clinical genomics filtering strategy can identify known driver genes and also reveal uncharacterized genes that may drive metastatic prostate cancer progression.

To validate our screen results, we demonstrated that eight top hits from this screen are required for prostate cancer cell proliferation or survival, suggesting our screen results and subsequent analysis nominate reproducible hit genes with a low false-positive rate (Fig. 1E). In order to demonstrate that the hits identified by these two integrated functional and clinical genomics approaches are potential prostate cancer-specific driver genes, we chose to study *KIF4A* and *WDR62* as they were relatively uncharacterized in the context of mCRPC.

### *KIF4A* is an AR-independent driver gene in metastatic prostate cancer

**Fig. 2:**
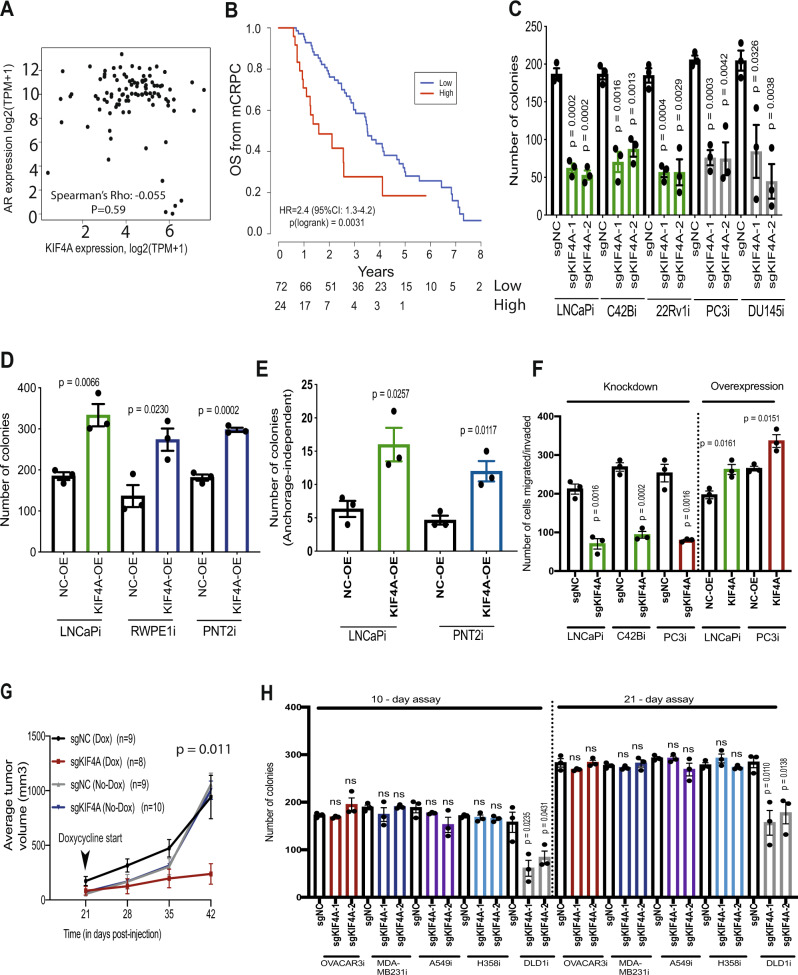
*KIF4A* is an AR-independent driver gene in metastatic prostate cancer. **A** Scatter plot showing no correlation between *AR* and *KIF4A* in 99 mCRPC patients based on a two-sided Spearman’s correlation test (Quigley et al); **B** A Kaplan–Meier curve of overall survival of 96 patients with CRPC with high and low level of *KIF4A*. Differences between groups were tested with a two-sided log-rank test. Hazard ratios were calculated using the Cox proportional hazards regression model. Number at risk is shown under the plot; **C** Colony formation assay in range of prostate cancer cell-line models with *KIF4A* knockdown (*n* = 3 as biological replicates; Mean ± SEM; Unpaired two-tailed *t*-test was used to determine statistical significance); **D** Colony formation assay in malignant and benign prostate cells with *KIF4A* overexpression (*n* = 3 as biological replicates; Mean ± SEM; Unpaired two-tailed *t*-test was used to determine statistical significance); **E** Anchorage-independent growth assay in malignant and benign prostate cells with *KIF4A* overexpression (*n* = 3 as biological replicates; Mean ± SEM; Unpaired two-tailed *t*-test was used to determine statistical significance); **F** Migration and Invasion assay with *KIF4A* knockdown and overexpression in malignant prostate cells (*n* = 3 as biological replicates; Mean ± SEM; Unpaired two-tailed *t*-test was used to determine statistical significance); **G** Line plot showing average tumor volume in *KIF4A* knockdown and control cells implanted in vivo. Average tumor volume was plotted and two-way ANOVA was used to measure statistical significance; **H** Colony formation assay in a range of non-prostate cancer CRISPRi cell-line models with *KIF4A* knockdown (*n* = 3 as biological replicates; Mean ± SEM; Unpaired two-tailed *t*-test was used to determine statistical significance).

We chose to further investigate *KIF4A* because two independent mCRPC clinical genomics datasets (Quigley, et al.^5^ and Abida, et al.^6^.) demonstrate that *KIF4A* is a copy number amplified in ~33% of mCRPC patient samples (Supplementary Fig. 4A, B) supporting the hypothesis that this may be a mCRPC driver gene. *KIF4A* and *AR* are both on the same arm of the X-chromosome and are frequently co-amplified despite being 2.55 megabases apart^5^. *AR* is also focally amplified in mCRPC^5^. The occurrence of recurrent long amplicons encompassing more than just *AR* supports the hypothesis that *KIF4A*, *AR*, and possibly additional genes may be a ‘cluster’ of cancer driver genes that are co-amplified on the X-chromosome to drive prostate cancer tumorigenesis. In localized primary human prostate cancer samples from the TCGA cohort, we observed that *KIF4A* and *AR* gene expression are correlated (Supplementary Fig. 5A). Surprisingly, in our mCRPC data^5^, we found no correlation between *AR* and *KIF4A* expression, suggesting an independent role for *KIF4A* from *AR* (Fig. 2A). In support of this finding, we also found no correlation between *AR* and *KIF4A* expression in another independent mCRPC cohort (Abida, et al.^6^.) (Supplementary Fig. 5B). Importantly, we found that high *KIF4A* expression was associated with poor outcome in mCRPC patients (Fig. 2B). There was also a strong positive correlation between expression of *KIF4A* and of *MKI67*, a marker of cell proliferation in mCRPC samples (in both Quigley et al. and Abida et al.) suggesting *KIF4A* is associated with cancer cell proliferation or survival in patients (Supplementary Fig. 6A, B). Collectively, these data suggest that *KIF4A* may have different functions in primary prostate cancer and mCRPC.

To experimentally dissect the role of *KIF4A* in prostate cancer, we tested whether *KIF4A* expression was required for prostate cancer cell proliferation and or survival in both AR-dependent (LNCaPi, C42Bi, and 22Rv1i) and AR-independent (PC3i and DU145i) cell-line models. We observed that inhibition of *KIF4A* expression reduced clonogenic survival in both AR-dependent and AR-independent prostate cancer models (Fig. 2C). *KIF4A* knockdown in LNCaPi cells did not decrease AR mRNA or protein levels (Supplementary Fig. 7A, B) *KIF4A* knockdown was similar across this panel of cell lines (Supplementary Fig. 8A). We also observed that *KIF4A* overexpression increased clonogenic survival in both malignant (LNCaPi) and benign prostate cells (RWPE1i and PNT2i) (Fig. 2D). *KIF4A* overexpression also increased anchorage-independent colony formation in both malignant and benign prostate cells (Fig. 2E). The level of KIF4A overexpression was similar across the cell-line models at mRNA level (Supplementary Fig. 8B) and at protein level (Supplementary Fig. 8C). Next, we modulated *KIF4A* expression and performed migration/invasion assays, and observed that the repression of *KIF4A* decreases cell migration/invasion while overexpression of *KIF4A* increases cell migration/invasion in AR-dependent as well as AR-independent cell models (Fig. 2F and Supplementary Fig. 8D). We also performed cell-cycle analysis on LNCaPi and PC3i cells, which are AR-dependent and -independent, respectively, with and without knockdown of *KIF4A*. *KIF4A* knockdown resulted in accumulation of cells in S phase (Supplementary Fig. 9A–D). Lastly, we created an inducible CRISPRi LNCaP (LNCaPi-Dox) model (Supplementary Figs. 10 and 11A). We implanted LNCaPi-Dox cells expressing negative control sgRNAs or sgRNAs targeting *KIF4A* cells into mice. At tumor onset, mice were treated with doxycycline to induce KIF4A knockdown or control. We observed that KIF4A is necessary for tumor growth in vivo (Fig. 2G). We collected tumor samples 21 days after addition of doxycycline and observed that KIF4A repression remained stable at this time point (Supplementary Fig. 11B).

Our clinical filtered screen results nominated *KIF4A* as a prostate-specific driver gene; however, DepMap CRISPR functional genomics data reported that *KIF4*A was essential in a number of cancer cell lines (Supplementary Fig. 3C). To test the model that *KIF4A* is a prostate-specific driver gene, we generated a panel of diverse CRISPRi non-prostate cancer cell-line models (breast: MDA-MB-231i, ovarian: OVCAR3i, lung: H358i and A549i, colon: DLD1i) (Supplementary Fig. 12A). We repressed *KIF4A* in each of these models and then measured the impact of *KIF4A* knockdown on cell proliferation and survival in 10-day and 21-day clonogenic survival assays. In five out of the six cell models, *KIF4A* was dispensable for cell proliferation or survival (Fig. 2H). Similarly, *KIF4A* knockdown in these non-prostate cancer cells did not show an effect on cell cycle (Supplementary Fig. 12C–H). *KIF4A* knockdown was similar across this panel of cell lines and also similar to *KIF4A* knockdown in prostate cancer cells (Supplementary Figs. 8A and 12B). Basal KIF4A protein levels do not correlate with gene essentiality across prostate and non-prostate cell-line models (Supplementary Fig. 13). This suggested that the driver gene properties of *KIF4A* could be relatively specific to prostate biology. Alternately, this discrepancy could be due to differences in the level of KIF4A disruption by CRISPR and by CRISPRi which would suggest that the exact level of KIF4A required for cell viability varies between cell types. If this is the case, then our data would demonstrate that there is a therapeutic window for inhibiting KIF4A as an anti-cancer strategy.

To further investigate why *KIF4A* is required for prostate cancer proliferation or survival, we measured the transcriptional consequences of *KIF4A* repression in LNCaPi cells. Using gene set enrichment analysis (GSEA), we observed a signature of multiple cancer signaling pathways, including *MYC* and *E2F*, known to drive cell proliferation (Supplementary Fig. 14A–E). *KIF4A* is a chromokinesin with two reported functions. *KIF4A* is reported to regulate multiple aspects of spindle organization and chromosome positioning/integrity, processes which are thought to be required for all dividing cells. KIF4A is also reported to form a complex with both DNMT3B^19^, an enzyme that catalyzes DNA methylation as well as key genes that regulate chromatin, such as HDAC1 and SIN3A^19^, suggesting KIF4A could be required for epigenetic programs that support mCRPC. To test whether repression of *KIF4A* alters the chromatin landscape of prostate cancer cells, we performed an Assay for Transposase-Accessible Chromatin using sequencing (ATAC-Seq) experiment in LNCaPi and C42Bi cells. Our data demonstrated that the patterns of open and closed chromatin are broadly remodeled upon *KIF4A* knockdown suggesting KIF4A may play a role in the regulation of chromatin biology in prostate cancer cells (Supplementary Figs. 15A–E and 16A, C). The altered ATAC-seq peaks were enriched for pathways which have been established to play crucial role in cancer cells proliferation and survival, including prostate cancer (Supplementary Fig. 16B, D).

### *WDR62* is an uncharacterized prostate cancer driver gene

**Fig. 3:**
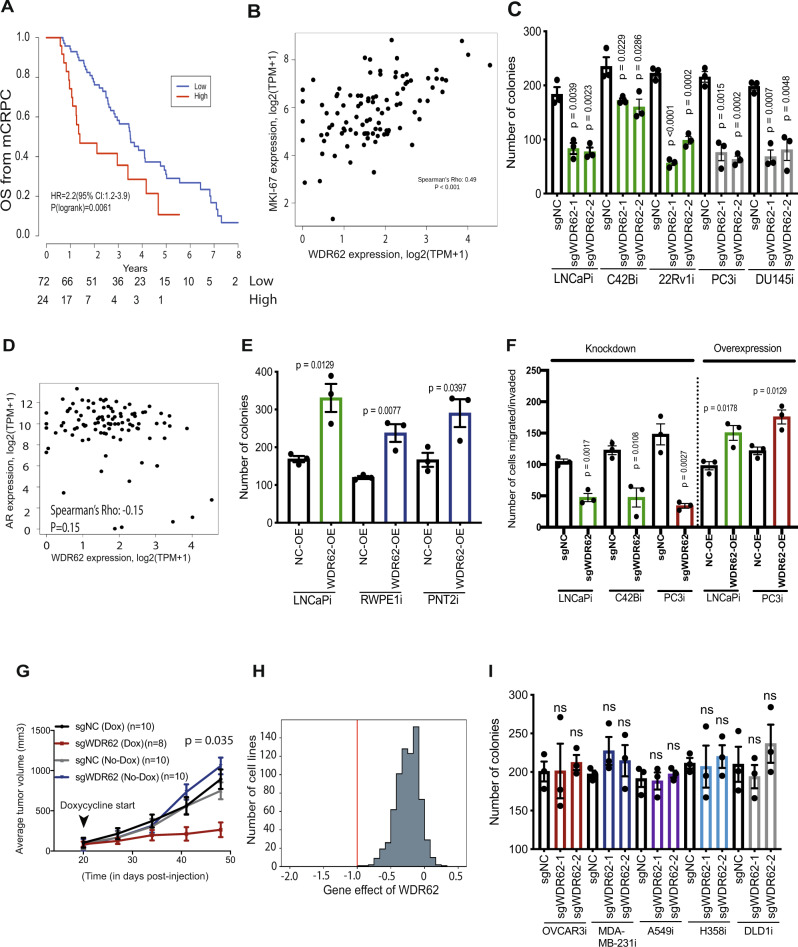
*WDR62* is an uncharacterized prostate cancer driver gene. **A** A Kaplan–Meier curve of overall survival of 96 patients with CRPC with high and low expression of *WDR62*^5^. Differences between groups were tested with a two-sided log-rank test. Hazard ratios were calculated using the Cox proportional hazards regression model. Number at risk is shown under the plot; **B** Scatter plot showing correlation between expression level of *MKI67* and *WDR62* in 99 mCRPC patients^5^. Spearman’s correlation with a two-sided test for significance was calculated; **C** Colony formation assay in a range of prostate cancer cell-line models with *WDR62* knockdown (*n* = 3 as biological replicates; Mean ± SEM; Unpaired two-tailed *t*-test was used to determine statistical significance); **D** Scatter plot (*n* = 99) showing no correlation between *WDR62* and *AR*^5^. Spearman’s correlation with two-sided test for significance was calculated; **E** Colony formation assay in malignant and benign prostate cells with *WDR62* overexpression (*n* = 3 as biological replicates; Mean ± SEM; Unpaired two-tailed *t*-test was used to determine statistical significance); **F** Migration and Invasion assay with *WDR62* knockdown and overexpression in malignant prostate cells (*n* = 3 as biological replicates; Mean ± SEM; Unpaired two-tailed *t*-test was used to determine statistical significance); **G** Line plot showing average tumor volume in *WDR62* knockdown and control cells implanted in vivo. Average tumor volume was plotted and two-way ANOVA was used to measure statistical significance; **H** Histogram of pan-cancer essentiality CERES scores of *WDR62* in DepMap database. The red line denotes the median gene effect of all common essential genes.; **I** Colony formation assay in a range of non-prostate cancer CRISPRi cell-line models with *WDR62* knockdown (*n* = 3 as biological replicates; Mean ± SEM; Unpaired two-tailed *t*-test was used to determine statistical significance).

We were intrigued by the observation that *WDR62* is an uncharacterized top CRISPRi screen hit gene that is crucial for prostate cancer cell proliferation or survival. In clinical genomics data, we observed that the expression of *WDR62* is significantly higher in primary (TCGA) and metastatic (MSKCC) prostate cancer samples relative to benign prostate samples (Supplementary Fig. 17A, B). We found that high *WDR62* expression is associated with poor patient outcome in mCRPC patients (Quigley et al.) (Fig. 3A). There is also a strong positive correlation between *WDR62* expression and *MKI67*, a marker of cell proliferation in mCRPC samples suggesting *WDR62* could drive tumor cell proliferation or survival (Fig. 3B). These data demonstrate that *WDR62* expression is correlated with disease progression and/or poor outcomes in mCRPC patients.

To experimentally dissect the role of WDR62 in prostate cancer, we performed clonogenic survival assays in five prostate cancer cell models and observed a significant decrease in the colony formation following knockdown of *WDR62* in all five models demonstrating *WDR62* is required for prostate cancer proliferation or survival irrespective of AR status (Fig. 3C). WDR62 basal protein expression and knockdown was similar across this panel of cell lines (Supplementary Fig. 18A, B). This is supported by our clinical data demonstrating *WDR62* levels are not correlated with *AR* levels in metastatic prostate cancer (Fig. 3D). Overexpression of *WDR62* significantly increased clonogenic survival of both malignant and benign prostate cells suggesting *WDR62* is a driver gene in prostate cancer that is likely independent of AR (Fig. 3E). The level of *WDR62* overexpression was similar across prostate malignant and benign cell-line models (Supplementary Fig. 18C). We also observed that knockdown of *WDR62* suppressed the ability of prostate cancer cells to migrate/invade, while *WDR62* overexpression increased migration and invasion (Fig. 3F). Lastly, we subcutaneously implanted LNCaPi-Dox cells expressing control sgRNAs or sgRNAs targeting *WDR62* into mice (Supplementary Fig. 19A). At tumor onset we treated the mice with doxycycline to induce *WDR62* knockdown and observed that WDR62 is required for tumor growth in vivo (Fig. 3G). Thirty days after addition of doxycycline, we collected the tumors and observed that WDR62 repression remains stable at that time point (Supplementary Fig. 19B). These results demonstrate *WDR62* is a driver of aggressive prostate cancer phenotypes both in vitro and in vivo.

Our functional genomics filters nominated *WDR62* as being selectively essential in prostate cancer models (Fig. 3H). However, to experimentally confirm this, we repressed *WDR62* expression in our panel of diverse CRISPRi non-prostate cancer cell-line models. In six out of six models, we found that *WDR62* is not required for cell proliferation and survival in a 10-day clonogenic survival assay or progression through the cell cycle (Fig. 3I, Supplementary Fig. 20A–D). In this experiment, WDR62 was repressed to an equivalent extent in each model as observed in CRISPRi prostate cell lines (Supplementary Fig. 20E). Collectively, these data suggest that *WDR62* is selectively essential in prostate cancer models. Basal protein expression of WDR62 was similar across this panel of non-prostate cancer cell lines (Supplementary Fig. 20F).

### WDR62 mediates the stability of the TPX2/AURKA protein complex in prostate cancer

**Fig. 4:**
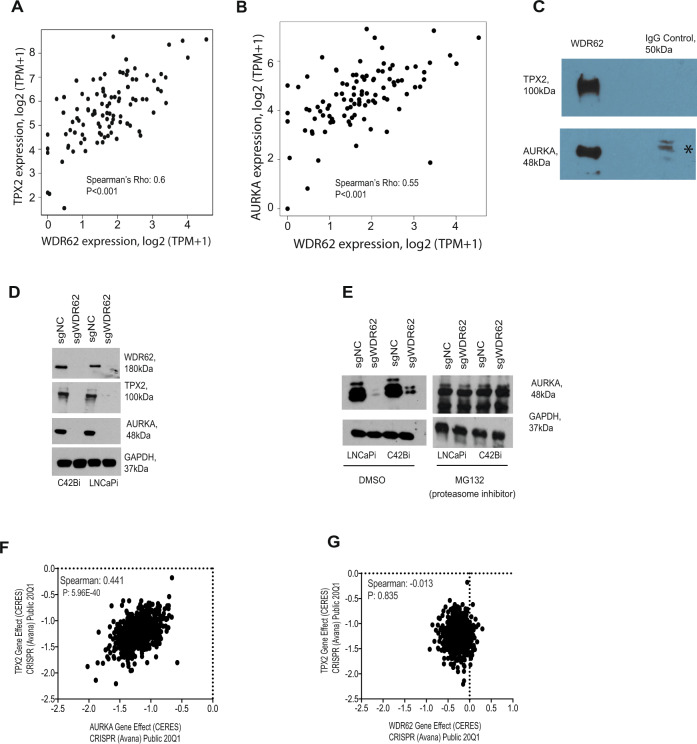
WDR62 mediates the stability of the TPX2/AURKA protein complex in prostate cancer. **A** and **B** Scatter plots (*n* = 99) showing a correlation between *TPX2* and *WDR62* and *AURKA* and *WDR62*, respectively^5^. Spearman’s correlation with a two-tailed test for significance was calculated; **C** Co-immunoprecipitation of WDR62 with TPX2 and AURKA (* non-specific band). The co-immunoprecipitation experiment was performed twice to determine reproducibility; **D** Western blot showing loss of AURKA and TPX2 following knockdown of WDR62. Each western blot experiment was performed twice to determine reproducibility; **E** Western blot of AURKA following knockdown of WDR62 with and without MG132, a proteasome inhibitor. Each western blot experiment was performed twice to determine reproducibility; **F** and **G** Scatter plots showing phenotype (gene effect) correlation between *TPX2* and *AURKA* and *TPX2* and *WDR62*, respectively^14^. Spearman’s correlation was performed for statistical analysis.

Relatively little is known about WDR62^20–25^. Mutations in WDR62 are associated with microcephaly in humans^20,22,23^. WDR62 has been reported to interact with Aurora A and TPX2^22,26^. However, this interaction is not annotated in protein–protein interaction databases^27^. In our mCRPC clinical data, we observed a strong positive correlation between *WDR62* and *TPX2* expression levels (Fig. 4A), as well as between *WDR62* and *AURKA* expression levels (Fig. 4B), which can reflect a functional relationship^28^. A similar correlation was also found in an independent mCRPC cohort (Abida et al.^6^) (Supplementary Fig. 21A, B). TPX2 and AURKA bind directly in a known protein complex that is required for mitosis in most cell types and as such both are common essential genes (Supplementary Fig. 21C, D)^29–31^. To test whether AURKA, TPX2, and WDR62 form a protein complex in mCRPC, we immunoprecipitated WDR62 from LNCaP cells and then western blotted for TPX2 and AURKA (Fig. 4C). This experiment demonstrated that WDR62 interacts with TPX2 and AURKA likely forming a protein complex in prostate cancer cells. We hypothesized that WDR62 may regulate the stability or function of the TPX2/AURKA protein complex in prostate cancer cells. We observed that the knockdown of WDR62 results in loss of TPX2 and AURKA protein suggesting WDR62 regulates the stability of this protein complex in prostate cancer cells (Fig. 4D). Mechanistically, we observed that upon knockdown of WDR62, AURKA is efficiently degraded by the proteasome (Fig. 4E).

To further confirm that WDR62 is a prostate cancer-specific regulator of the TPX2/AURKA protein complex, we examined *WDR62*, *AURKA*, *TPX2* genetic dependencies in the DepMap. As expected, *TPX2* and *AURKA* genetic dependencies are highly correlated (Fig. 4F). In contrast, there is no correlation between *WDR62* and *TPX2* phenotypes in DepMap data compatible with the hypothesis that *WDR62* specifically regulates *TPX2/AURKA* in prostate cancer cells (Fig. 4G). Notably, only a single prostate cancer cell line (VCaP) is represented in the DepMap data and so we do not expect a pan-cancer correlation between *TPX2* and *WDR62* if the biology of *WDR62* is prostate cancer-specific. Together, these data suggest that WDR62 is a prostate cancer-specific driver gene and in addition is a potential therapeutic target in mCRPC.

## Discussion

Most advanced cancers are driven by the biochemical activity of multiple driver genes. In prostate cancer, AR is the major driver of disease progression, but it remains unclear how additional genes drive mCRPC through AR-dependent and independent mechanisms. In addition, an emerging theme in mCRPC is that not all tumors are addicted to AR^32–34^. Using a genome-scale CRISPRi functional genomics platform, we have identified genes required for proliferation or survival in two models of advanced prostate cancer. Integrative analysis of these data with clinical genomics data and functional genomics data revealed AR as a top hit in these screens but also nominated a number of additional poorly characterized prostate-specific driver genes. Our work highlights the strength of an integrated clinical and functional genomics pipeline and focuses on two of the poorly characterized hit genes. Our data demonstrated *KIF4A* and *WDR62* are AR-independent prostate cancer driver genes that are associated with poor prognosis in patients with advanced metastatic disease.

Our interest in *KIF4A* was driven by the clinical data analysis showing this gene has many of the properties of a human cancer driver gene. In support of this, our data in vitro and in vivo data demonstrated that *KIF4A* promotes phenotypes associated with poor prognosis in mCRPC, irrespective of AR status, suggesting *KIF4A* is a driver gene that promotes disease progression in human prostate cancers. Mechanistically, KIF4A biology is complex and the role of KIF4A in prostate cancer is fairly unknown. We demonstrated through transcriptome profiling and genome-wide chromatin accessibility analysis that knockdown of *KIF4A* reveals gene signatures that are established to have profound effect on prostate cancer phenotypes. However, open questions remain with respect to how KIF4A interacts with other proteins to modulate gene expression, chromatin biology, and prostate cancer phenotypes. We note that KIF4A has been nominated as a clinical biomarker of aggressive prostate cancer^35^. Our data currently does not support the hypothesis that KIF4A and AR form an auto-regulatory positive feedback loop in prostate cancer^36^. However, it will be important to model KIF4A in genetically engineered mouse models of prostate cancer to further explore the biology of how KIF4A promotes tumorigenesis, metastasis, and drug response.

A top hit in our CRISPRi screen is *WDR62*, an uncharacterized gene in prostate cancer. The identification of *WDR62* and additional hit genes as poorly characterized prostate cancer driver genes highlights the strength of our integrated clinical and functional genomics analysis strategy. *WDR62* is one of the most commonly mutated autosomal recessive primary microcephaly-associated genes, with over 30 identified mutations leading to reduced brain size and a spectrum of cortical abnormalities^20,37^. In this study, we demonstrated that WDR62 is highly expressed in primary as well as metastatic prostate cancer and is associated with poor patient outcome. We demonstrated both in vitro and in vivo that *WDR62* promotes aggressive prostate cancer phenotypes in all of the prostate cancer models that were tested, irrespective of AR-status, suggesting that this is an AR-independent human prostate cancer driver gene. We note that WDR62 was previously characterized as a biomarker of resistance to AR targeted therapies in models of neuroendocrine prostate cancer (NEPC) and in NEPC patient samples however to our knowledge WDR62 has not been previously implicated as a driver of prostate cancer adenocarcinoma^38^. Mechanistically, our data demonstrated that WDR62 modulates the activity of AURKA by stabilizing the AURKA/TPX2 protein complex in prostate cancer cells. Genetically engineered mouse models of prostate cancer are needed to further explore the biology of how *WDR62* promotes tumorigenesis, metastasis, and drug response. *AURKA* and *TPX2* are required for proliferation in most cells; however, our data and data from DepMap demonstrated that *WDR62* is uniquely essential for proliferation in prostate cancer cells, suggesting *WDR62* could be a potential molecular target in mCRPC patients to disrupt *AURKA*-*TPX2* signaling without being lethal to other non-prostate tissues^39–41^. However, additional studies are required to prove that WDR62 does not regulate AURKA/TPX2 activity across other cell types.

To our knowledge, no previous studies have utilized the strengths of integrating clinical genomics and functional genomics data to identify prostate cancer-specific driver genes. Our manuscript leverages an integrated clinical and functional genomics pipeline to identify and validate genes that can drive aggressive prostate cancer phenotypes. As expected, *AR* was the top hit in our analysis. Development of novel agents targeting mCRPC drivers, other than AR, such as KIF4A and WDR62 may provide important therapeutic strategies for mCRPC patients. Furthermore, for patients with metastatic AR-dependent prostate cancer innovative combination therapies that co-target multiple driver genes simultaneously may lead to increased patient survival.

## Methods

### Cell culture

LNCaP, C42B, 22Rv1, PC3, DU145, RWPE-1, OVCAR3, MDA-MB-231, A549, H358, DLD1 cell lines were purchased from ATCC. PNT2 cells were purchased from Millipore Sigma. RPMI 1640 medium (Gibco Catalog number: 11875119) + 10% FBS (fetal bovine serum) were used to grow all the cell lines, except RWPE-1 and HEK293T cells. RWPE-1 cells were grown using Keratinocyte Serum Free Medium (Gibco Catalog number: 17005042) + 10% FBS. HEK293T cells were grown using DMEM medium (Gibco Catalog number: 11885084) + 10% FBS. All cell lines underwent verification by short tandem repeat profiling at Genetica Cell-Line Testing.

### CRISPR vectors

Lentiviral vectors were used to express the CRISPRi protein and sgRNAs in human cells as previously described^10^. The CRISPRi protein (dCas9-BFP-KRAB) was expressed from either the SFFV or TET3G promoter. The sgRNA vector encodes an sgRNA driven by the mouse U6 promoter as well as a fluorescent protein (BFP or GFP) T2A puromycin N-acetyl transferase gene driven by the human EF1α promoter. The sgRNA sequences used in experiments are provided in Supplementary Table 2.

Lentivirus generation: HEK293T cells were used to generate lentivirus. pCMV-dR8.91 and pMD2-G were used as packing vectors. LT1 transfection reagent (Mirus MIR2300) was used for transfection. To enhance viral production viral boost reagent (Alstem # VB100) was used.

### Cell-line construction

For constitutive and inducible CRISPRi cell lines, polyclonal cells expressing dCas9-BFP-KRAB fusion proteins driven from an SFFV or TRE3G promoter, respectively, were generated by viral transduction followed by fluorescence-activated cell sorting to purity using a BD Fusion. Prostate cancer CRISPRi lines are denoted as LNCaPi, C42Bi, 22Rv1i, PC3i, and DU145i. LNCaPi, C42Bi, and 22Rv1i AR-dependent prostate cancer lines, whole PC3i and DU145i are AR-independent cell lines. Benign prostate CRISPRi lines are denoted as RWPE1i and PNT2i. Doxycycline inducible CRISPRi line on LNCaP line is denoted as LNCaPi-Dox. Non-prostate cancer CRISPRi lines are denoted as OVCAR3i (is an ovarian cancer line), MDA-MB-231i (is a breast cancer line), A549i and H358i (are lung cancer lines), and DLD1i (is a colon cancer line).

### CRISPRi screen

The human CRISPRi V2 Top5 sgRNA library was used to perform CRISPRi screens^11^. This sgRNA library targets 18,905 human genes with five sgRNAs per transcription start site. Cells were grown at minimum library coverage of 1000x for genome-scale screens. Cells were collected at 0 and 8 doubling after puromycin selection and harvested cells were processed for next-generation sequencing. The screens were performed in two technical replicates. Briefly, DNA was isolated, the cassette encoding the sgRNA was amplified by PCR, and relative sgRNA abundance was determined by next-generation sequencing as previously described^10,11^. Data were analyzed using publicly available code (https://github.com/mhorlbeck/ScreenProcessing).

### Clinical cohorts

Quigley et al.^5^ is the published whole-genome and -transcriptome analysis of 101 castration-resistant prostate cancer metastases. Gene expression and copy number data were processed as described in the original publication. Abida et al.^6^ TCGA^42^ and MSKCC^43^ datasets were downloaded through the cBioPortal for Cancer Genomics^44,45^, and gene expression and copy number data were analyzed as processed by cBioPortal. All analyses on these cohorts were performed in the R statistical environment version 3.6.3 using RStudio 1.2.503^46,47^. The correlation was calculated by Spearman’s rank correlation. A two-sided Wilcoxon rank-sum test was used to test for differences between two groups, unless otherwise stated. Violin plots were created using the ggplot2 package^48^. Survival analyses for the Quigley et al. study were performed with overall survival as endpoint, calculated from the time of mCRPC diagnosis until death or censoring, and visualized using the Kaplan–Meier method using the survival package and plotting functions from the rms package^49–52^. Differences between groups were tested with a two-sided log-rank test. Hazard ratios were calculated using the Cox proportional hazards regression model. High expression was defined as the top quartile of gene expression for the respective genes.

### Clinical genomics filters

Considering that drivers of mCRPC commonly have copy number gains^6,7^, first, we ranked the genes (*n* = 1472) by number of mCRPC samples having a copy number gain of the gene in the Quigley et al. We then further narrowed down this list of genes by the criteria that a copy number gain would result in a corresponding change in phenotype (gene expression) due to the copy number gain. The cut-off for the change in gene expression with or without copy number gain was set to be ≥ 2 fold change in mean expression (transcripts per million, TPM) with *p* value ≤ 0.001 (two-sided Wilcoxon rank-sum test).

### Combined functional genomics and clinical data filters

The goal of this filter was to identify prostate cancer-specific genes that are expressed higher in metastases compared to primary prostate tumors and/or benign prostate. First, the 1472 genes were filtered against the published pan-cancer, except prostate cancer PICKLES database^15^. Genes that were called as essential in 90% of the cell lines were removed from the gene list. Second, the gene list was filtered through the CERES cancer dependency database (DepMap, Version 20Q1)^13^. Genes called essential by the DepMap database were removed from the list. Third, we further removed any gene on the list that were found to be as a significant hit in two non-prostate cancer CRISPRi screen published recently^16^. Finally, the genes whose expression were found to be high in metastasis samples (in MSKCC cohort) compared to primary tumor were only considered. The narrowed-down gene list was then ranked based on their screen phenotype score and *p* value (smallest to largest).

For DepMap gene effect data, as defined by DepMap a score of 0 is equivalent to a gene that is not essential, whereas a score of −1 corresponds to the median of all common essential genes^14,53,54^.

### Competitive growth assay

LNCaPi cells plated out at 150,000 cells per well in six-well plates were infected with lentivirus sgRNA targeting gene of interest and control. Each lentiviral transduction of sgRNA and control were done in three biological replicates and performed at least twice to determine the reproducibility of the data. Data were collected and analyzed using Invitrogen Attune NxT Flow Cytometer at day 3 post-infection and every 48 h thereafter. The relative fraction of sgRNA in the infection cells was measured.

### Clonogenic assay

Cells transduced with the indicated sgRNAs and then were treated with puromycin for achieve a pure population of the transduced cells. One thousand cells were seeded out per well in a six-well plate. Each experimental setup was performed in three biological replicates and done at least thrice to determine the reproducibility of the data. RNA was isolated from the remaining cells to confirm knockdown by qRT-PCR. The cells were allowed to grow for 4 days. On day 5, the wells were washed with PBS, and colonies are fixed with 25% methanol and stained with crystal violet (0.05% w/v). Images were scanned and analyzed using ImageJ software. Average number of colonies formed was counted and two-tailed *t*-test was used to determine statistical significance.

### Migration and invasion assay

Cells transduced with sgRNAs targeting the gene of interest and control were treated with puromycin for a pure population. A total of 100,000 cells were then plated in serum-free media for invasion in Matrigel-coated micropore transwells (Corning Bio-Coat). Each experimental setup was performed in three biological replicates and done at least thrice to determine the reproducibility of the data. RNA was isolated from the remaining cells to confirm knockdown by qRT-PCR. Three days after invasion/migration plating, transwells were fixed in 4% formaldehyde, stained with crystal violet, and cells on the upper side of the micropore membrane wiped off. Cells on the lower side of the membrane were photographed with a microscope, the dye was dissolved in 10% acetic acid and optical density measured at 560 nm. Average number of cells migrated was counted and two-tailed *t*-test was used to determine statistical significance.

### Overexpression vectors

Mammalian gene collection fully sequenced human KIF4A cDNA (Horizon, Cat #MHS6278-202758261) and human WDR62 (Horizon, Cat #MHS6278-202830915) was used to transfect cells and overexpress KIF4A and WDR62, respectively. Overexpression efficiency was measure either by qPCR or by western blotting.

### Soft-agar assay

Growth in anchorage-independent condition was assessed by colony formation in low melting agarose. Melted agar solution (1%) was plated out in 10 cm plates and allowed to solidify. Cells transduced to have gene overexpression and control were counted and prepared. Each experimental setup was performed in three biological replicates and done at least thrice to determine the reproducibility of the data. The cell suspension was mixed with 0.6% low melting agar (1:1) and plated out over the solidified agar. The cells were allowed to grow for 21 days. The colonies were then stained with nitroblue tetrazolium chloride. Images were scanned and analyzed using ImageJ software. Average number of colonies formed was counted and two-tailed *t*-test was used to determine statistical significance.

### Cell-cycle assay

Stably expressing dCas9-KRAB CRISPRi cells were plated out at 150,000 cells per well and lentiviral transfected with sgRNA targeting KIF4A and control sgRNA. Post puromycin selection, cells were stained with manufacturers protocol^55^. Briefly, harvested cells were washed with PBS, counted, fixed with 70% ethanol and stained with Propidium Iodide Solution (Cat# 421301). Each experimental setup was performed in three biological replicates and done at least thrice to determine the reproducibility of the data.

### In vivo experiments

Doxycycline inducible LNCaP cells were lentiviral transfected with sgRNA targeting KIF4A (and also WDR62) and control sgRNA. Post puromycin selection, cells were counted, and the cell suspension was mixed with Matrigel (1:1). A total of 2 × 10^6^ cells were injected subcutaneously on each flank of the mice. NOD-SCID-Gamma mice were used. UCSF IACUC and laboratory animal resource center (LARC) mouse husbandry standards were followed for all mouse housing (https://larc.ucsf.edu/). Once the tumors were palpable the mice were randomly categorized in two groups receiving either doxycycline diet (Bio-Serv, Cat#S3888) or control diet. Tumors were measured using digital caliper by two different laboratory personnel to rule out any measurement bias. Tumor volume was calculated using the equation: Volume = length × width^2^ × 0.52, where the length represents the longer axis. Average tumor volume was plotted and two-way ANOVA was used to measure statistical significance. The mice were humanely euthanized once the tumor reached 1000 cubic mm of size following appropriate UCSF’s LARC protocol. Protein was extracted from the tumors of the mice to determine knockdown efficiency. All animal experiments conducted were reviewed and approved by UCSF IACUC board.

### RNA extraction and qPCR

Cells were viral transduced with sgRNA targeting the gene of interest or control sgRNA for 3 days and selected with puromycin for pure population for 3 days. The entire setup of the experiment was done in three biological replicates. Post-selection RNA was extracted from the cells as per the manufacturer’s protocol using the Zymo Quick-RNA-extraction kit Cat# R1054. RNA quantification was done using Thermo Scientific Nanodrop 2000 system. cDNA was prepared using SuperScript III First-Strand Synthesis System for RT-PCR (Cat # 18080-051). QuantStudio Flex Real-Time PCR system was used to measure mRNA expression of gene of interest. The list of all primers used is provided in Supplementary Table 1.

### Western blot

Cells were lysed using RIPA buffer with protease inhibitor (Thermo, Cat#78430), sonicated and centrifuged to extract protein. Either NuPAGE 4–12% Bis-Tris or 3-8%Tris-Acetate precast polyacrylamide gels were used for protein analysis^56,57^. Antibodies used were as follows: KIF4A (Thermo, Cat# PA5-30492), WDR62 (Bethyl Labs, Cat#A301-560A), GAPDH (14C10) (Cell signaling, Cat#2118), Aurora A (D3E4Q) (Cell signaling, Cat #14475), TPX2 (Novus Bio, Cat#NB500-179), AR (D6F11) (Cell Signaling #5153). Antibody specificity for primary antibodies was validated by CRISPRi knockdown experiments. All antibodies were raised for human proteins and used to detect human proteins. All the western blot experiments were at least done twice to determine reproducibility.

### RNA-Seq sample preparation

LNCaPi cells were viral transduced with sgRNA targeting KIF4A or control sgRNA for 3 days and selected with puromycin for pure population for 3 days. Post-selection RNA was extracted from the cells as described above for library preparation to generate Illumina compatible libraries of sequences and to perform qPCR to determine the sgRNA knockdown efficiency. QuantSeq 3′mRNA-Seq library prep kit FWD for Illumina (Lexogen, Cat# 015.24) was used to prepare the library as per the manufacturer’s protocol. Quality control was performed by using the Agilent Bioanalyzer 2100 system and the samples were sequenced in Illumina HiSeq 4000. The entire set of the experiment was done in two biological replicates.

### RNA-seq data processing

The RNA-seq single-end fastq data generated by Illumina HiSeq 4000 sequencing system were first trimmed to remove adapter sequence using Cutadapt v2.6^58^ with “-q 10 -m 20” option. After adapter trimming, FASTQC v0.11.8^59^ was used to evaluate the sequence trimming as well as overall sequence quality. Using splice-aware aligner STAR (2.7.1a)^60^, RNA-seq reads were aligned onto the Human reference genome build hg38 using “–outSAMtype BAM SortedByCoordinate–outSAMunmapped Within–outSAMmapqUnique 50–sjdbOverhang 65–chimSegmentMin 12–twopassMode Basic” option and exon-exon junctions, according to the known Human gene model annotation from the GENCODE v30^61^. Apart from protein-coding genes, non-coding RNA types, and pseudogenes are further annotated and classified. Furthermore, based on the reads that can only be mapped to a single genomic location, the transcript/gene expression quantification was performed using “featurecount” function within Rsubread R-package^62^ with “GTF.featureType = “exon”, GTF.attrType = “gene_id”, useMetaFeatures = TRUE, allowMultiOverlap = FALSE, countMultiMappingReads = FALSE, isLongRead = FALSE, ignoreDup = FALSE, strandSpecific = 0, juncCounts = TRUE, genome = NULL, isPairedEnd = FALSE, requireBothEndsMapped = FALSE, checkFragLength = FALSE, countChimericFragments = TRUE, autosort = TRUE” option. Cross-sample normalization of expression values and differential expression analysis between the KIF4A-knockdown and control was done using DESeq2 R-package^63^. Benjamini-Hochberg corrected p-value < 0.05 and log2 fold change >0.5 or <0.5 were considered statistically significant.

The RNA-seq data generated in this study have been deposited in the NCBI’s Gene Expression Omnibus (GEO) database under accession code GSE178330.

### ATAC-Seq sample preparation

We performed ATAC-Seq on LNCaP and C42B cells following knockdown of KIF4A or control. The experiment was carried out as described in the published method papers by Buenrostro, et al^64^. and Corces, et al^65^. with the following modifications. Cells were resuspended in buffer (Illumina Cat#), incubated on ice for 10 min, and lysed using a dounce homogenizer. 50,000 nuclei were incubated with 25uL 2X TD Buffer and 1.25uL Transposase (Illumina Tagment Enzyme/Buffer Cat# 20034210) shaking at 300 rpm at 37 C for 30 min. Zymo DNA Clean and Concentrator 5 kit (Cat# D4014) was then used to purify DNA. Transposed DNA was amplified using PCR master mix and indexes from Nextera DNA Library Prep kit (Cat# 15028211) for 5 cycles and then assessed using qPCR. Final cleanup was performed using 1.8X AMPure XP beads (Cat# A63881) and libraries quantified using the DNA High Sensitivity Agilent 2100 Bioanalyzer System. Samples were sequenced at the UCSF Core Facility on the NovaSeq, paired end. The entire experimental setup was performed in two technical replicates.

### ATAC-seq data processing

The ATAC-seq paired-end fastq data generated by Illumina NovaSeq 6000 sequencing system were first trimmed to remove Illumina Nextera adapter sequence using Cutadapt v2.6^58^ with “-q 10 -m 20” option. After adapter trimming, FASTQC v0.11.8^59^ was used to evaluate the sequence trimming as well as overall sequence quality. Bowtie2 version 2.3.5.1^66^ was then used to align the ATAC-seq reads against the Human reference genome build hg38 using “–very-sensitive” option. The uniquely mapped reads were obtained in SAM format. Samtools version 1.9^67^ was used to convert SAM to BAM file as well as sort the BAM file. Picard (https://broadinstitute.github.io/picard/) was then used to remove duplicates using the MarkDuplicates tool using “REMOVE_DUPLICATES = true” option. The resulting BAM file reads position were then corrected by a constant offset to the read start (“+” stranded +4 bp, “-” stranded −5 bp) using deepTools2 v3.3.2^68^ with “alignmentSieve–ATACshift” option. This resulted in the final aligned, de-duplicated BAM file that was used in all downstream analyses. ATAC-seq peak calling was performed using MACS2 v2.2.5^69^ to obtain narrow peaks with “callpeak -f BAMPE -g hs–nomodel -B–keep-dup all–call-summits” option. The resulting peaks that map to the mitochondrial genome or genomic regions listed in the ENCODE hg38 blacklist (https://www.encodeproject.org/annotations/ENCSR636HFF/) or peaks that extend beyond the ends of chromosomes were filtered out. Non-overlapping unique ATAC-seq narrow peaks regions were obtained from the all samples analyzed. Only those non-overlapping unique peak regions present in at least two samples were considered for further analysis. Sequencing reads mapped to these non-overlapping unique regions were counted using “featurecount” function within Rsubread R-package^62^ with “isPairedEnd = −TRUE, countMultiMappingReads = FALSE, maxFragLength = 100, autosort = TRUE” option. Further normalization of the feature counts and differential open chromatin regions between KIF4A-knockdown and control were obtained using DESeq2 R-package^63^. Only those peak regions with Benjamini-Hochberg corrected *p*-value < 0.05 and log2 foldchange >0.5 or <0.5 were considered statistically significant.

The ATAC-seq data generated in this study have been deposited in the NCBI’s GEO database under accession code GSE178330 (https://www.ncbi.nlm.nih.gov/geo/query/acc.cgi?acc=GSE178330)

### Gene set enrichment analysis (GSEA)

We used GSEA^70^ to identify the signaling pathways enriched in the differentially expressed genes between KIF4A-knockdown and control obtained from RNA-seq analysis. For this, we used fgsea R-package^71^ with Hallmark pathway collection from MSigDB^72^.

### Statistical analysis

In the Quigley et al. study, Spearman’s correlation was used to determine statistical significance for all the correlation plots: *KIF4A* and *AR*; *MKI67* and *KIF4A*; *WDR62* and *AR*; *MKI67* and *WDR62*. For gene expression and correlation, a two-sided Wilcoxon rank-sum test was used to test for differences between two groups, unless otherwise stated. Survival analyses for the Quigley et al. study were performed with overall survival as endpoint, calculated from time of mCRPC diagnosis until death or censoring, and visualized using the Kaplan–Meier method. Differences between groups were tested with a two-sided log-rank test. Hazard ratios were calculated using the Cox proportional hazards regression model. Spearman’s or Pearson correlation analysis was used for all other datasets: prostate cancer TCGA, Abida et al. and MSKCC.

Unpaired *t*-test was used to determine statistical analysis for all the column plots: colony formation assay, anchorage-independent assay, migration and invasion assay, and qPCR results. Two-way ANOVA was used to determine statistical significance in the in vivo data. Spearman’s correlation analysis was performed a correlation of CERES data between TPX2 and AURKA and TX2 and WDR62. In RNA-Seq data, Benjamini–Hochberg test was performed. Corrected *p* value < 0.05 and log2 foldchange >0.5 or <0.5 were considered statistically significant. In ATAC-Seq data peak regions with Benjamini-Hochberg corrected *p*-value < 0.05 and log2 foldchange >0.5 or <0.5 were considered statistically significant.

### Reporting summary

Further information on research design is available in the Nature Research Reporting Summary linked to this article.

## Supplementary information

Supplementary Information

Description of Additional Supplementary Files

Supplementary Data 1

Supplementary Data 2

Supplementary Data 3

Supplementary Data 4

Reporting Summary

## Data Availability

All data generated or analyzed during this study are included in this published article and its supplementary information files. The RNA sequencing and ATAC sequencing datasets generated in this study have been deposited in the NCBI’s Gene Expression Omnibus (GEO) database under accession code GSE178330. PICKLES (http://pickles.hart-lab.org) and DepMap (https://depmap.org/portal/) data are publicly available. A reporting summary for this article is available as a Supplementary Information file.

## Code availability

Custom code used in this manuscript is publicly available at https://github.com/GilbertLabUCSF/CanDI or freely available on request.

## References

[1] Siegel RL, Miller KD, Jemal A (2019). Cancer statistics, 2019. CA Cancer J. Clin..

[2] Massie CE (2011). The androgen receptor fuels prostate cancer by regulating central metabolism and biosynthesis. EMBO J..

[3] Meng MV (2002). Contemporary patterns of androgen deprivation therapy use for newly diagnosed prostate cancer. Urology.

[4] Kirby M, Hirst C, Crawford ED (2011). Characterising the castration-resistant prostate cancer population: a systematic review. Int. J. Clin. Pract..

[5] Quigley DA (2018). Genomic hallmarks and structural variation in metastatic prostate cancer. Cell.

[6] Abida W (2019). Genomic correlates of clinical outcome in advanced prostate cancer. Proc. Natl Acad. Sci. USA.

[7] Robinson D (2015). Integrative clinical genomics of advanced prostate cancer. Cell.

[8] Tyner J. W. Integrating functional genomics to accelerate mechanistic personalized medicine. *Cold Spring Harb. Mol. Case Stud*. https://www.ncbi.nlm.nih.gov/pmc/articles/PMC5334473/ (2017). 10.1101/mcs.a001370 PMC5334473 28299357

[9] Behan FM (2019). Prioritization of cancer therapeutic targets using CRISPR–Cas9 screens. Nature.

[10] Gilbert LA (2014). Genome-scale CRISPR-mediated control of gene repression and activation. Cell.

[11] Horlbeck M. A. et al. Compact and highly active next-generation libraries for CRISPR-mediated gene repression and activation. eLife **5**, e19760 (2016). 10.7554/eLife.19760 PMC5094855 27661255

[12] Comparative genomic and transcriptomic analyses of LNCaP and C4-2B prostate cancer cell lines. https://journals.plos.org/plosone/article?id=10.1371/journal.pone.0090002 (2020). 10.1371/journal.pone.0090002 PMC3938550 24587179

[13] Tsherniak A (2017). Defining a cancer dependency map. Cell.

[14] Meyers RM (2017). Computational correction of copy-number effect improves specificity of CRISPR-Cas9 essentiality screens in cancer cells. Nat. Genet..

[15] Lenoir WF, Lim TL, Hart T (2018). PICKLES: the database of pooled in-vitro CRISPR knockout library essentiality screens. Nucleic Acids Res..

[16] Lou K., et al. KRASG12C inhibition produces a driver-limited state revealing collateral dependencies. *Sci. Signal.* https://www.ncbi.nlm.nih.gov/pmc/articles/PMC6871662/ (2019). 10.1126/scisignal.aaw9450 PMC6871662 31138768

[17] Ewing CM (2012). Germline mutations in HOXB13 and prostate-cancer risk. N. Engl. J. Med..

[18] Norris JD (2009). The homeodomain protein HOXB13 regulates the cellular response to androgens. Mol. Cell.

[19] Geiman TM (2004). Isolation and characterization of a novel DNA methyltransferase complex linking DNMT3B with components of the mitotic chromosome condensation machinery. Nucleic Acids Res..

[20] Nicholas AK (2010). WDR62 is associated with the spindle pole and is mutated in human microcephaly. Nat. Genet..

[21] Alshawaf A. J., Antonic A., Skafidas E., Ng D. C.-H., Dottori M. WDR62 regulates early neural and glial progenitor specification of human pluripotent stem cells. *Stem Cells Int.* https://www.hindawi.com/journals/sci/2017/7848932/ (2017). 10.1155/2017/7848932 PMC5485354 28690640

[22] Chen J-F (2014). Microcephaly disease gene Wdr62 regulates mitotic progression of embryonic neural stem cells and brain size. Nat. Commun..

[23] Jayaraman D (2016). Microcephaly proteins Wdr62 and aspm define a mother centriole complex regulating centriole biogenesis, apical complex, and cell fate. Neuron.

[24] Shohayeb B (2020). The association of microcephaly protein WDR62 with CPAP/IFT88 is required for cilia formation and neocortical development. Hum. Mol. Genet..

[25] Qin Y. et al. WDR62 is involved in spindle assembly by interacting with CEP170 in spermatogenesis. *Development*. http://dev.biologists.org/content/146/20/dev174128 (2019). 10.1242/dev.174128 31533924

[26] Lim NR (2015). Opposing roles for JNK and Aurora A in regulating the association of WDR62 with spindle microtubules. J. Cell Sci..

[27] Szklarczyk D (2019). STRING v11: protein–protein association networks with increased coverage, supporting functional discovery in genome-wide experimental datasets. Nucleic Acids Res..

[28] Taggart JC, Li G-W (2018). Production of protein-complex components is stoichiometric and lacks general feedback regulation in eukaryotes. Cell Syst..

[29] Kufer TA (2002). Human TPX2 is required for targeting Aurora-A kinase to the spindle. J. Cell Biol..

[30] Fu J, Bian M, Liu J, Jiang Q, Zhang C (2009). A single amino acid change converts Aurora-A into Aurora-B-like kinase in terms of partner specificity and cellular function. Proc. Natl Acad. Sci. USA.

[31] Huttlin EL (2015). The BioPlex network: a systematic exploration of the human interactome. Cell.

[32] Beltran H (2016). Divergent clonal evolution of castration-resistant neuroendocrine prostate cancer. Nat. Med.

[33] Bluemn EG (2017). Androgen receptor pathway-independent prostate cancer is sustained through FGF signaling. Cancer Cell.

[34] Liu Y., et al. The androgen receptor regulates a druggable translational regulon in advanced prostate cancer. *Sci. Transl. Med.* https://stm.sciencemag.org/content/11/503/eaaw4993 (2019). 10.1126/scitranslmed.aaw4993 PMC6746573 31366581

[35] Gao H, Chen X, Cai Q, Shang Z, Niu Y (2018). Increased KIF4A expression is a potential prognostic factor in prostate cancer. Oncol. Lett..

[36] Cao Q (2020). Targeting the KIF4A/AR axis to reverse endocrine therapy resistance in castration-resistant prostate cancer. Clin. Cancer Res..

[37] Bilgüvar K (2010). Whole-exome sequencing identifies recessive WDR62 mutations in severe brain malformations. Nature.

[38] Lin D (2014). High fidelity patient-derived xenografts for accelerating prostate cancer discovery and drug development. Cancer Res..

[39] Beltran H (2019). A Phase II trial of the Aurora kinase A inhibitor alisertib for patients with castration-resistant and neuroendocrine prostate cancer: efficacy and biomarkers. Clin. Cancer Res. J. Am. Assoc. Cancer Res..

[40] Beltran H (2011). Molecular characterization of neuroendocrine prostate cancer and identification of new drug targets. Cancer Discov..

[41] Kivinummi K (2017). The expression of AURKA is androgen regulated in castration-resistant prostate cancer. Sci. Rep..

[42] Cancer Genome Atlas Research Network. The molecular taxonomy of primary prostate cancer. *Cell*. **163**, 1011–1025 (2015). 10.1016/j.cell.2015.10.025 PMC4695400 26544944

[43] Taylor BS (2010). Integrative genomic profiling of human prostate cancer. Cancer Cell.

[44] Cerami E (2012). The cBio cancer genomics portal: an open platform for exploring multidimensional cancer genomics data. Cancer Discov..

[45] Gao J (2013). Integrative analysis of complex cancer genomics and clinical profiles using the cBioPortal. Sci. Signal..

[46] RStudio | Open source & professional software for data science teams https://rstudio.com/ (2021).

[47] R: the R project for statistical computing https://www.r-project.org/ (2021).

[48] Welcome | ggplot2 https://ggplot2-book.org/ (2021).

[49] Therneau T. M. Survival analysis [R package survival version 3.2-11]. Comprehensive R Archive Network (CRAN). https://CRAN.R-project.org/package=survival (2021).

[50] Borgan Ø. Modeling survival data: extending the Cox model. (eds. Therneau a, T. M. & Grambsch, P. M.) (Springer-Verlag, New York, 2000). No. of pages: xiii + 350. ISBN 0-387-98784-3.

[51] rms: Regression modeling strategies. Comprehensive R Archive Network (CRAN). https://CRAN.R-project.org/package=rms (2021).

[52] Ghandi M (2019). Next-generation characterization of the Cancer Cell Line Encyclopedia. Nature.

[53] Yogodzinski C., Arab A., Pritchard J. R., Goodarzi H., Gilbert L. A. A global cancer data integrator reveals principles of synthetic lethality, sex disparity and immunotherapy. Preprint at *bioRxiv* https://www.biorxiv.org/content/10.1101/2021.01.08.425918v1.full (2021). 10.1186/s13073-021-00987-8 PMC8524992 34663427

[54] Pacini C (2021). Integrated cross-study datasets of genetic dependencies in cancer. Nat. Commun..

[55] Protocol - propidium iodide cell cycle staining protocol. https://www.biolegend.com/en-us/protocols/propidium-iodide-cell-cycle-staining-protocol (2020).

[56] Das R (2017). MicroRNA-194 promotes prostate cancer metastasis by inhibiting SOCS2. Cancer Res..

[57] Paltoglou S (2017). Novel androgen receptor co-regulator GRHL2 exerts both oncogenic and anti-metastatic functions in prostate cancer. Cancer Res..

[58] Martin M (2011). Cutadapt removes adapter sequences from high-throughput sequencing reads. EMBnet. J..

[59] Andrews S. FastQC: A quality control tool for high throughput sequence data http://www.bioinformatics.babraham.ac.uk/projects/fastqc/ (2010).

[60] Dobin A (2013). STAR: ultrafast universal RNA-seq aligner. Bioinformatics.

[61] Frankish A (2019). GENCODE reference annotation for the human and mouse genomes. Nucleic Acids Res..

[62] Liao Y, Smyth GK, Shi W (2019). The R package Rsubread is easier, faster, cheaper and better for alignment and quantification of RNA sequencing reads. Nucleic Acids Res..

[63] Love MI, Huber W, Anders S (2014). Moderated estimation of fold change and dispersion for RNA-seq data with DESeq2. Genome Biol..

[64] Buenrostro J., Wu B., Chang H., Greenleaf W. ATAC-seq: a method for assaying chromatin accessibility genome-wide. *Curr. Protoc. Mol. Biol.* **109**, 21.29.1–21.29.9 (2015). 10.1002/0471142727.mb2129s109 PMC4374986 25559105

[65] Corces MR (2017). An improved ATAC-seq protocol reduces background and enables interrogation of frozen tissues. Nat. Methods.

[66] Langmead B, Salzberg SL (2012). Fast gapped-read alignment with Bowtie 2. Nat. Methods.

[67] Li H (2009). The Sequence Alignment/Map format and SAMtools. Bioinformatics.

[68] Ramírez F (2016). deepTools2: a next generation web server for deep-sequencing data analysis. Nucleic Acids Res..

[69] Zhang Y (2008). Model-based analysis of ChIP-Seq (MACS). Genome Biol..

[70] Subramanian A (2005). Gene set enrichment analysis: a knowledge-based approach for interpreting genome-wide expression profiles. Proc. Natl Acad. Sci. USA.

[71] Korotkevich G., Sukhov V., Sergushichev A. Fast gene set enrichment analysis. Preprint at *bioRxiv* https://www.biorxiv.org/content/10.1101/060012v3 (2019).

[72] Liberzon A (2015). The molecular signatures database (MSigDB) hallmark gene set collection. Cell Syst..

