# Supplementary material for: An integrated functional and clinical genomics approach reveals genes driving aggressive metastatic prostate cancer
Source: Nat Commun. 2021 Jul 29;12:4601. doi: 10.1038/s41467-021-24919-7 (PMC8322386; doi:10.1038/s41467-021-24919-7)
Supplement: Supplementary file 1 — Supplementary Information [file 41467_2021_24919_MOESM1_ESM.pdf]

Supplementary Figure 1: CRISPRi cell line validation by qPCR using sgRNAs targeting *DPH2* and *ST3GAL4* (noted in the figure as *ST3)*, two previously validated CRISPRi positive control genes, on a range of malignant and benign prostate lines (n=3 as biological replicates; Mean ± SEM; Unpaired two-tailed t-test was used to determine statistical significance).

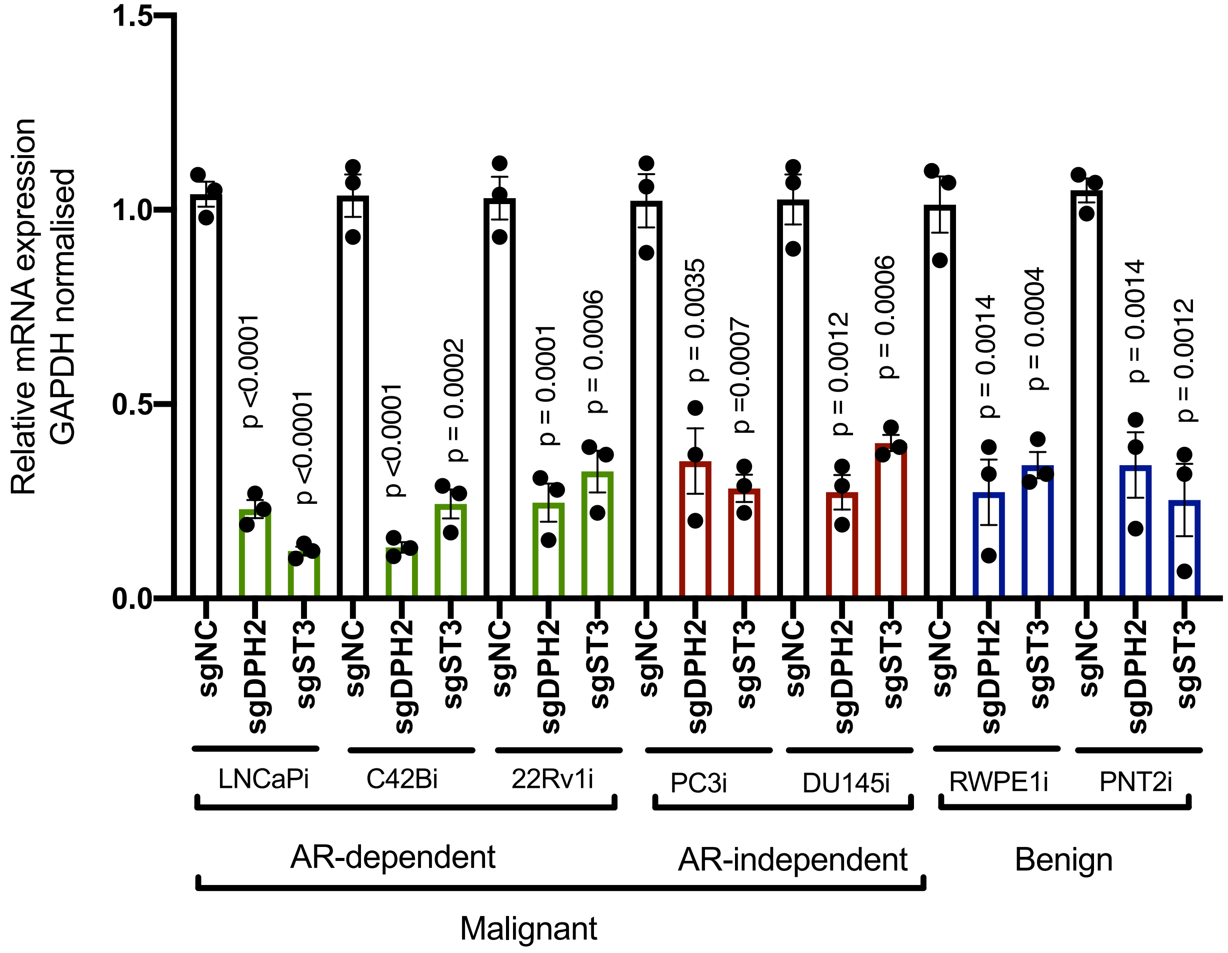

Supplementary Figure 2: Supplementary Figure 2A and 2B are the scatter plots showing correlation between independent replicate LNCaPi and C42Bi screens, at sgRNA level or gene level respectively. Genes or sgRNAs targeting protein coding genes are shown in black while the non-targeting sgRNA pseudogenes or individual sgRNAs are shown in grey. Supplementary Figure 2C is a Venn diagram showing number of genes shared between and selective to LNCaPi and C42Bi screens.

A. Correlation between replicates of LNCaPi screens at sgRNA level

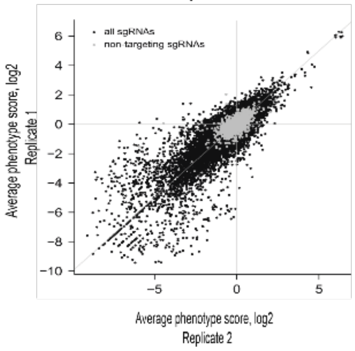

B. Correlation between replicates of C42Bi screens at gene level

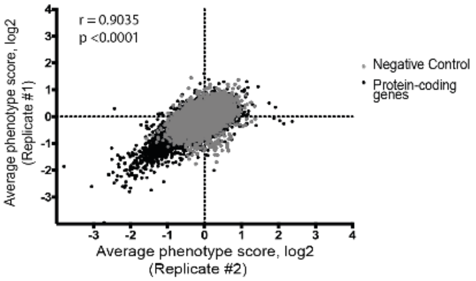

C. Number of genes shared between and selective to LNCaPi and C42Bi screens

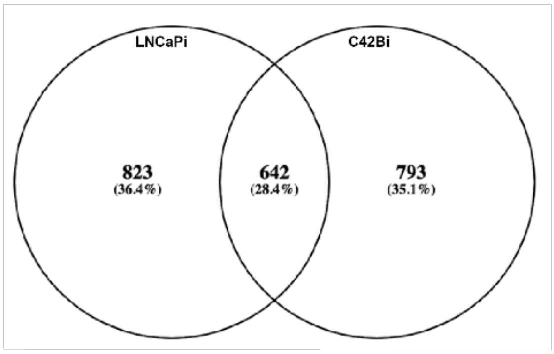

.

Supplementary Figure 3: Histograms of pan-cancer essentiality CERES scores in DepMap database for the 5 top hits nominated by the clinical genomics filters. (A) AR, (B) TSR2, (C) KIF4A, (D) MRPL13 and (E) NDUFB11. The red line denotes the median gene effect of all common essential genes.

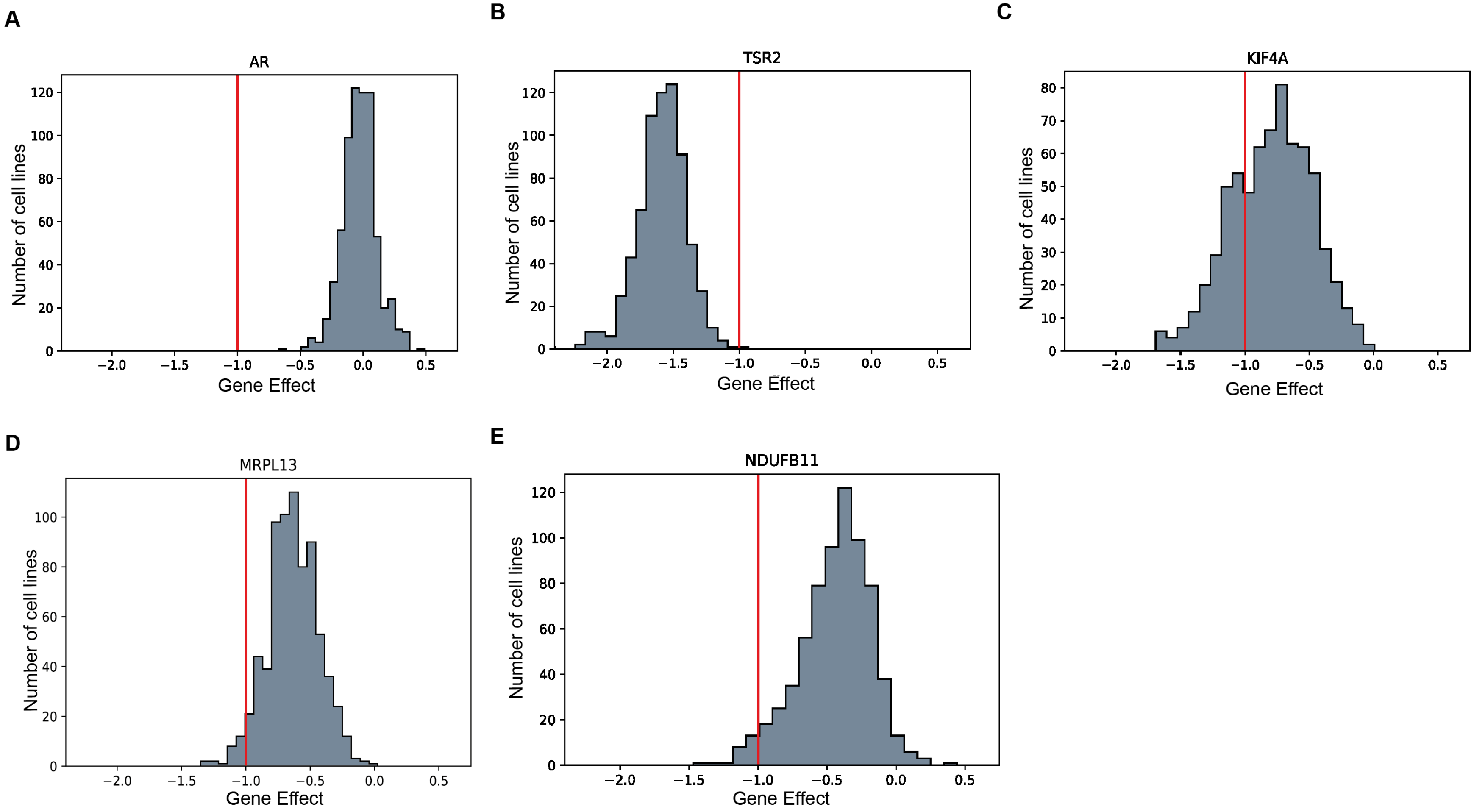

Supplementary Figure 4: Amplification of *KIF4A* in mCRPC patient samples. (A) Violin and box plot showing expression levels with (n=33) or without (n=66) copy number gain of KIF4A in Quigley, et al. A two-sided Wilcoxon rank-sum test was performed to test for statistical significance. Boxplot shows median with hinges at 25th and 75th percentiles and whiskers at largest/smallest value within 1.5 * inter quartile range. Individual data points are plotted as points together with the "violin" as a mirrored density plot. (B) Bar diagram showing copy number amplification with gain of KIF4A in Abida, et al. (n=444).

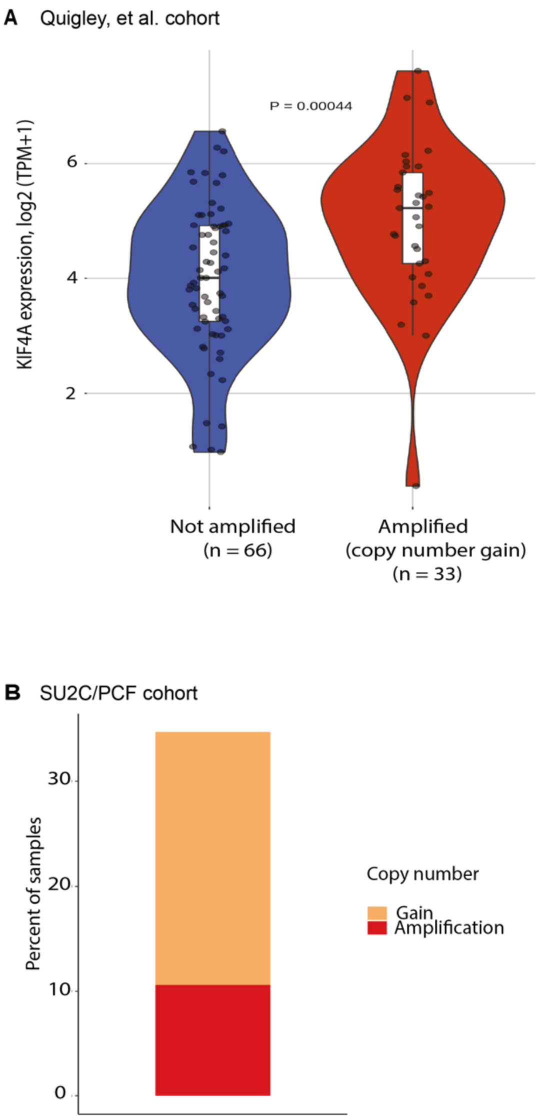

Supplementary Figure 5: Scatter plot showing (A) correlation between *KIF4A* and *AR* in prostate cancer TCGA cohort and (B) no correlation between *KIF4A* and *AR* in Abida, et al. study. Spearman's and Pearson's correlation were performed for statistical analysis.

A Prostate TCGA cohort (333 primary prostate tumors)

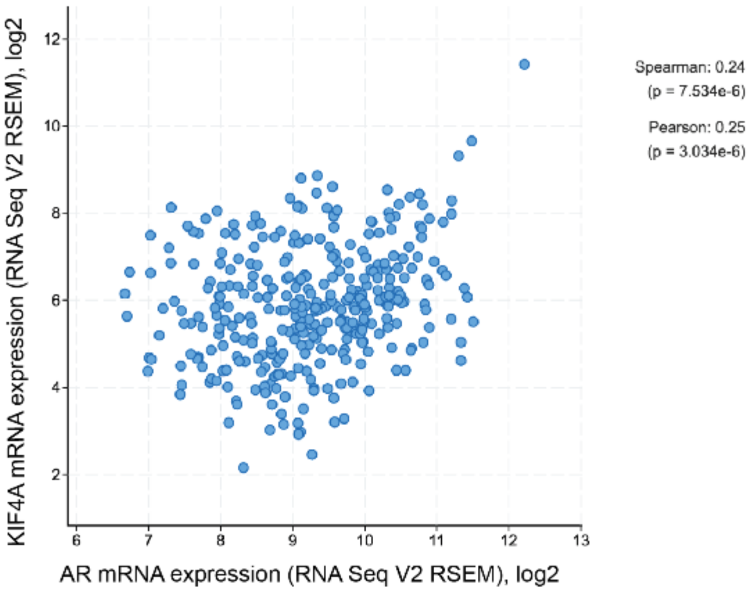

B SU2C/PCF mCRPC cohort (444 tumors: 14 primary prostate tumors and 430 metastasis)

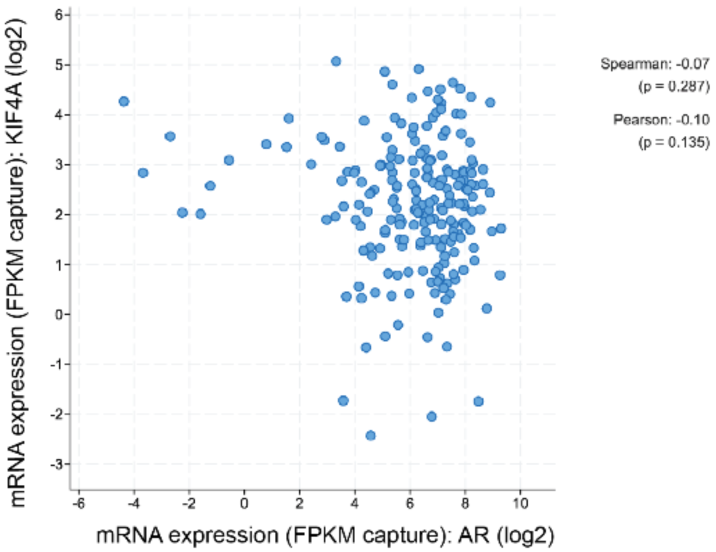

Supplementary Figure 6: Scatter plot showing correlation between *MKI-67* and *KIF4A* expression in two independent mCRPC cohorts. (A) For Quigley, et al. (n=99) Spearman's correlation with two-sided test for significance was calculated. (B) For Abida, et al. Spearman's and Pearson's correlation were performed for statistical analysis.

A Quigley, et al. cohort

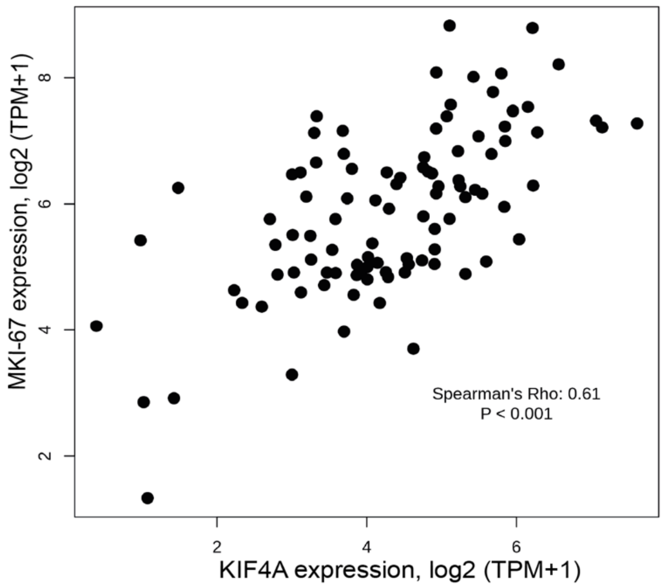

B SU2C/PCF cohort

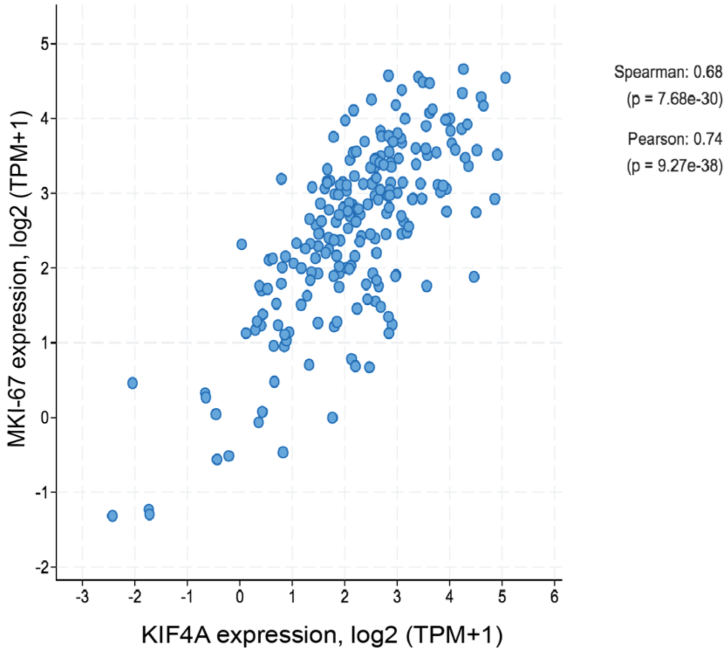

Supplementary Figure 7: (A) A graph showing the expression of AR at mRNA level measured by qPCR (n=3 as biological replicates; Mean ± SEM; Unpaired two-tailed t-test was used to determine statistical significance. (B) A western blot showing AR protein level following knockdown of KIF4A knockdown in LNCaP cells. Western blot experiment was performed twice to determine reproducibility.

A

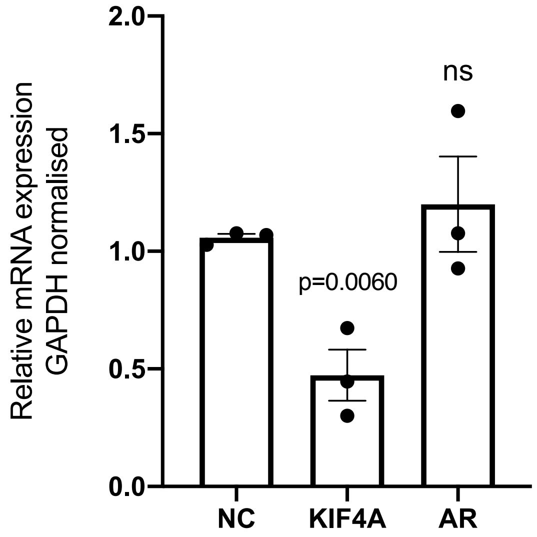

B

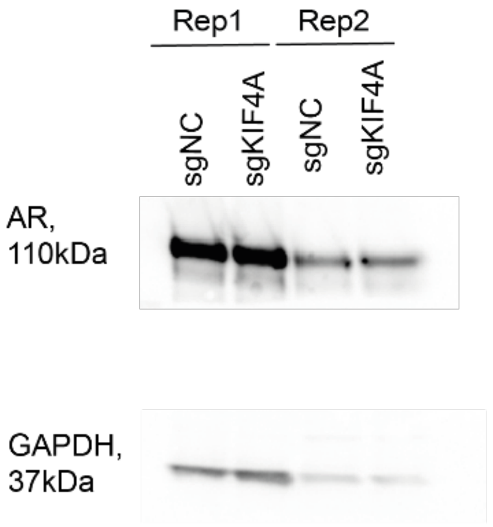

Supplementary Figure 8: A graph showing (A) *KIF4* knockdown efficiency in CRISPRi prostate cancer cell-line models and (B) A graph showing *KIF4A* cDNA overexpression in parental malignant and benign prostate cell line models, measured by qPCR (n=3 as biological replicates; Mean ± SEM; Unpaired two-tailed t-test was used to determine statistical significance). (C) Western blot showing KIF4A cDNA overexpression in LNCaP and C42B cells. Western blot experiment was performed twice to determine reproducibility. (D) Scratch wound assay assessing the migratory capability of LNCaP cells following KIF4A knockdown. The experiment was done with n = 6 as biological replicates for each sgRNA group (sgNC and sgKIF4A). Statistical significance was calculated using Mann-Whitney test (non-parametric test) at 24 and 48 hours. * $p<0.05$.

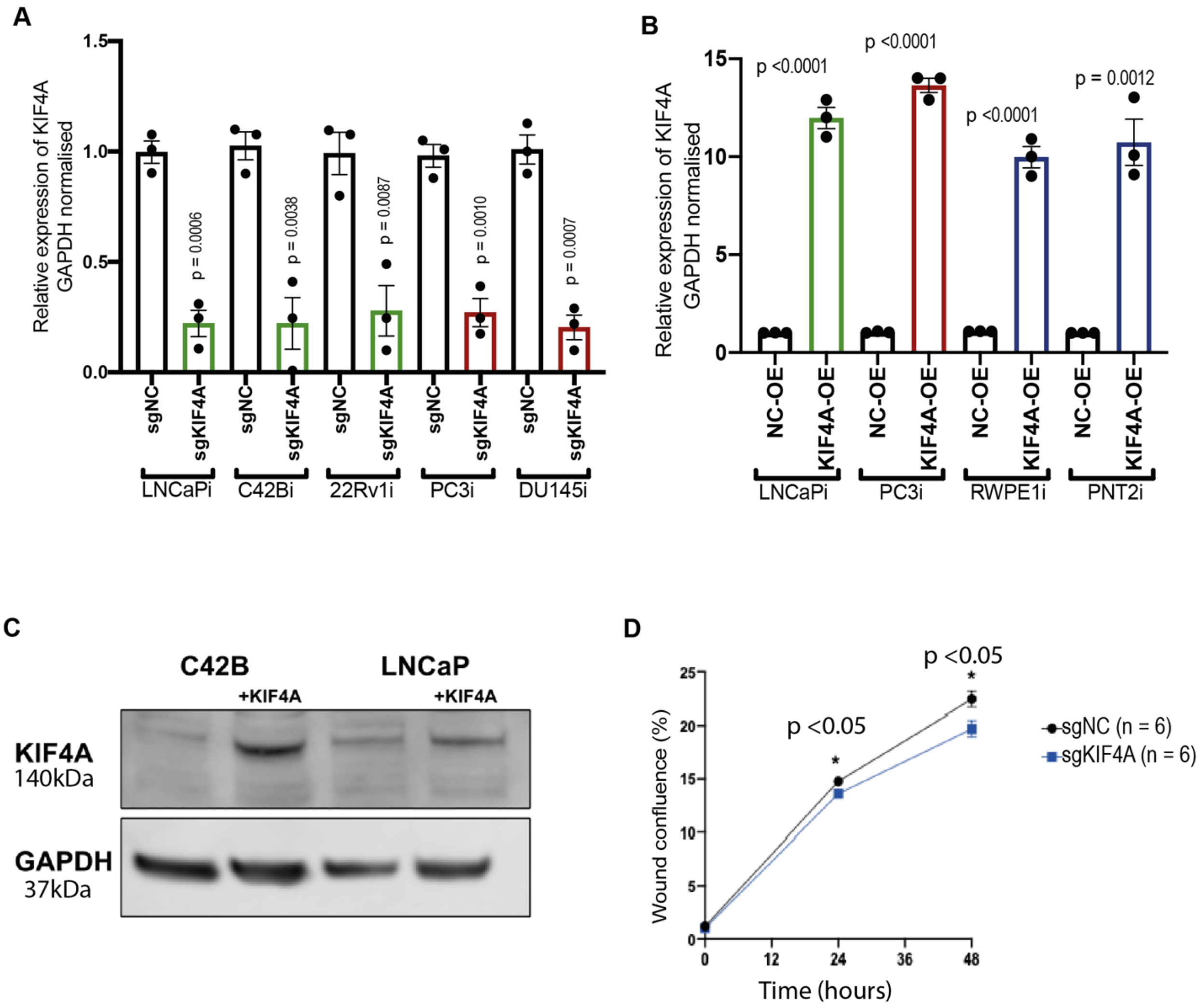

Supplementary Figure 9: Flow cytometry analysis of the effect of *KIF4A* knockdown relative to controls on cell cycle in two prostate cancer CRISPRi cell-line models, (A) LNCaPi and (B) PC3i. (C) A table showing the raw values of the cell cycle assays performed in LNCaPi and PC3i cells with KIF4A knockdown and control (n = 2 biological replicates for each cell-line model). (D) Figure showing FACS sequential gating strategies used for cell-cycle analyses.

A

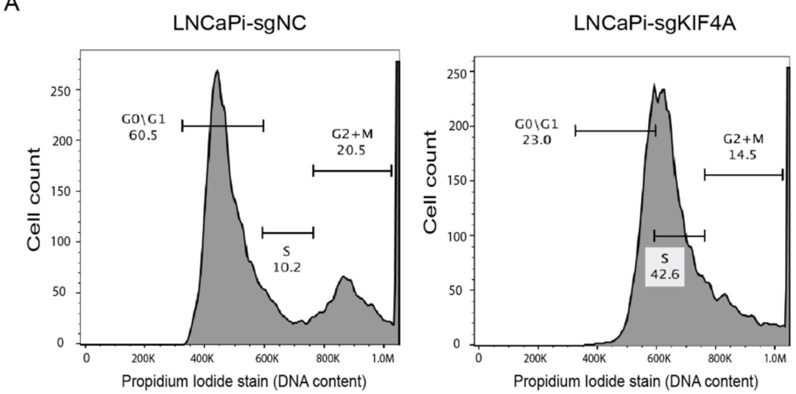

B

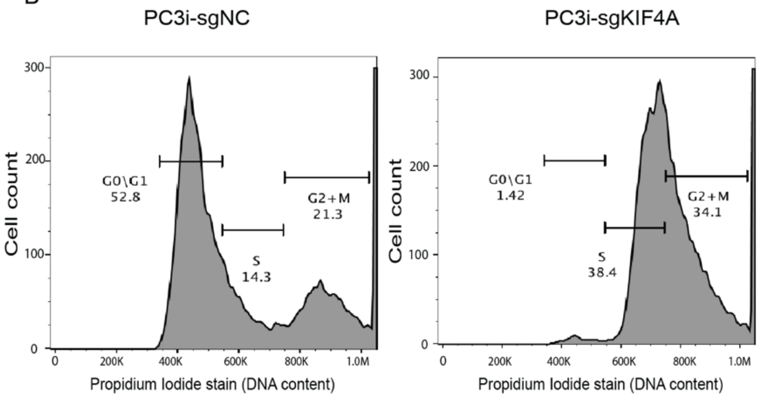

C

Raw values	LNCaPi -sgNC						LNCaPi –sgKIF4A					
	G0\G1 (%)	SEM	S (%)	SEM	G2+M (%)	SEM	G0\G1 (%)	SEM	S (%)	SEM	G2+M (%)	SEM
Rep # 1	60.5	6.85	10.2	1.78	20.5	4.05	23.0	1.55	42.6	1.25	14.5	0.65
Rep # 2	74.2		6.63		12.4		26.1		45.1		13.2	
	PC3i-sgNC						PC3i-sgKIF4A					
	G0\G1 (%)	SEM	S (%)	SEM	G2+M (%)	SEM	G0\G1 (%)	SEM	S (%)	SEM	G2+M (%)	SEM
Rep # 1	52.8	3.15	14.3	2.15	21.3	3.35	1.42	0.59	38.4	1.25	34.1	0.80
Rep # 2	59.1		10.0		14.6		0.23		35.9		35.7	

D

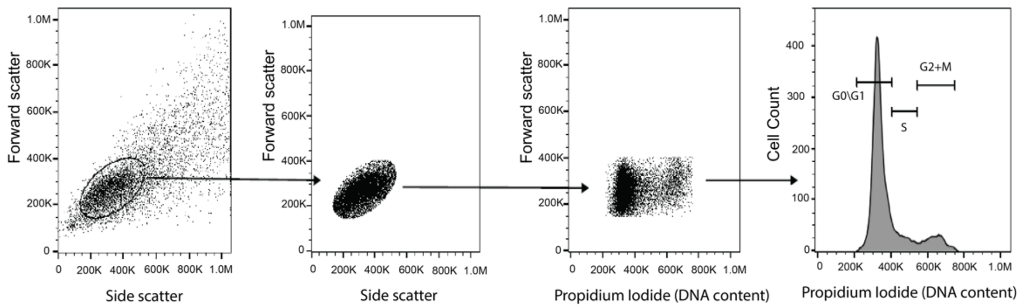

Supplementary Figure 10: Validation of doxycycline inducible LNCaPi-Dox cells with sgRNAs targeting *DPH2* and *ST3GAL4* (noted in the figure as *ST3)*, two positive control CRISPRi genes, measured by qPCR *in vitro* (n=3 as biological replicates; Mean ± SEM; Unpaired two-tailed t-test was used to determine statistical significance.

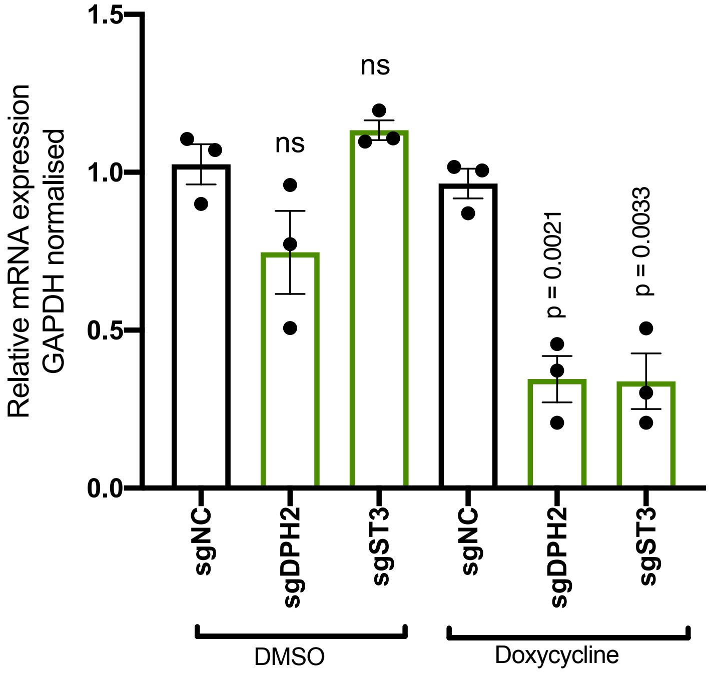

Supplementary Figure 11: Knockdown efficiency of KIF4A in LNCaPi-Dox cells. (A) *In vitro* assessment of *KIF4A* knockdown relative to control measured by qPCR *in vitro* before the subcutaneous implantation of the cancer cells (n=3 as biological replicates; Mean + SEM; Unpaired two-tailed t-test was used to determine statistical significance. (B) Knockdown efficiency of KIF4A relative to control measured at protein level extracted from the tumors from 6 different mice after 21days post-implantation. Western blot experiment was performed twice to determine reproducibility.

A

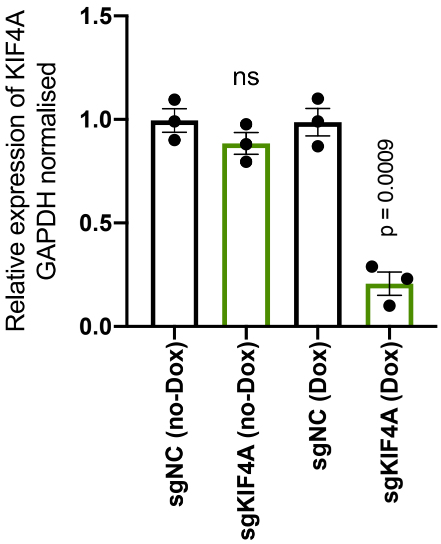

B

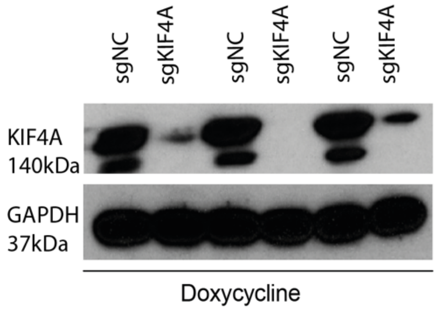

Supplementary Figure 12: Non-prostate cancer CRISPRi cell-line models. (A) Validation of the lines by qPCR using sgRNAs targeting *DPH2* and *ST3GAL4* (n=3 as biological replicates; Mean ± SEM; Unpaired two-tailed t-test for significance values. (B) Knockdown efficiency of *KIF4A* in a range of non-prostate cancer cell-line models, measured by qPCR (n=3 as biological replicates; Mean± SEM; Unpaired two-tailed t-test was used to determine statistical significance. Flow cytometry analysis of the effect of *KIF4A* knockdown with control on cell cycle in non-prostate cancer CRISPRi cell line models, (C) MDA-MB-231i, (D) OVCAR3i, (E) A549i, (F) DLD1i and (G) H358i, respectively. (H) A table showing the raw values of the cell cycle assays performed in MDA-MD-231i, OVCAR3i, A549i, DLD1i and H358i cells with KIF4A knockdown and control (n = 2 biological replicates for each cell- line model).

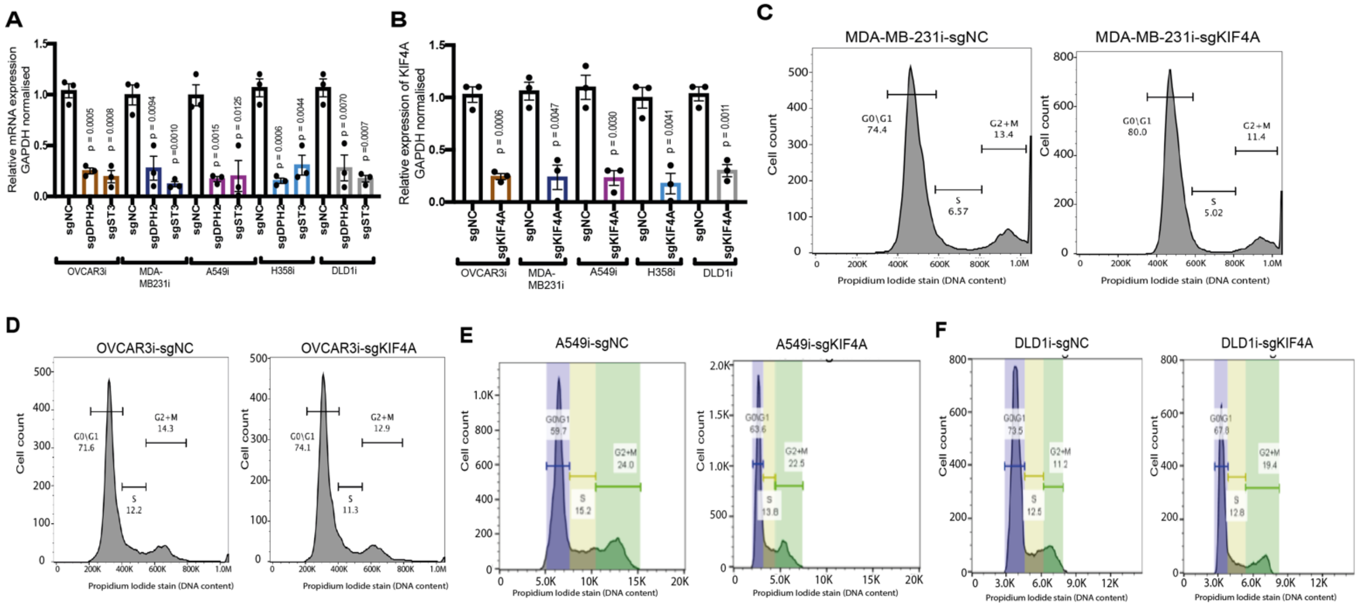

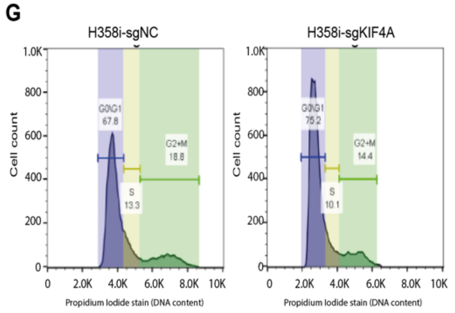

H

Raw values	MDA-MB-231i -sgNC						MDA-MB-231i -sgKIF4A					
	G0\G1 (%)	SEM	S (%)	SEM	G2+M (%)	SEM	G0\G1 (%)	SEM	S (%)	SEM	G2+M (%)	SEM
Rep # 1	74.2	3.55	6.57	2.66	13.4	1.9	80.0	3.6	5.02	1.44	11.4	1.1
Rep # 2	67.1		11.9		17.2		72.8		7.9		13.6	
	OVACAR3i-sgNC						OVCAR3i-sgKIF4A					
	G0\G1 (%)	SEM	S (%)	SEM	G2+M (%)	SEM	G0\G1 (%)	SEM	S (%)	SEM	G2+M (%)	SEM
Rep # 1	71.6	3.4	12.2	0.05	14.3	3.05	74.1	1.9	11.3	1.1	12.9	2.7
Rep # 2	64.8		12.3		20.4		70.3		13.5		18.3	
	H358i -sgNC						H358i -sgKIF4A					
	G0\G1 (%)	SEM	S (%)	SEM	G2+M (%)	SEM	G0\G1 (%)	SEM	S (%)	SEM	G2+M (%)	SEM
Rep # 1	67.8	4.1	13.3	1.4	18.8	2.55	75.2	2.1	10.1	1.48	14.4	2.1
Rep # 2	76.0		10.5		13.7		71.0		7.14		10.2	
	A549i -sgNC						A549i -sgKIF4A					
	G0\G1 (%)	SEM	S (%)	SEM	G2+M (%)	SEM	G0\G1 (%)	SEM	S (%)	SEM	G2+M (%)	SEM
Rep # 1	59.7	4.2	15.2	1.1	24.0	1.7	63.6	2.7	13.8	0.95	22.5	2.2
Rep # 2	68.1		17.4		27.4		69.0		11.9		18.1	
	DLD1i-sgNC						DLD1i-sgKIF4A					
	G0\G1 (%)	SEM	S (%)	SEM	G2+M (%)	SEM	G0\G1 (%)	SEM	S (%)	SEM	G2+M (%)	SEM
Rep # 1	73.5	11.3	12.5	1.45	11.2	2.45	67.8	1.2	12.8	2.55	19.4	3.75
Rep # 2	96.1		15.4		16.1		70.2		17.9		11.9	

Supplementary Figure 13: Basal expression of KIF4A in two benign and five malignant prostate cell line models and five non-prostate cancer cell line models. Western blot experiment was performed twice to determine reproducibility.

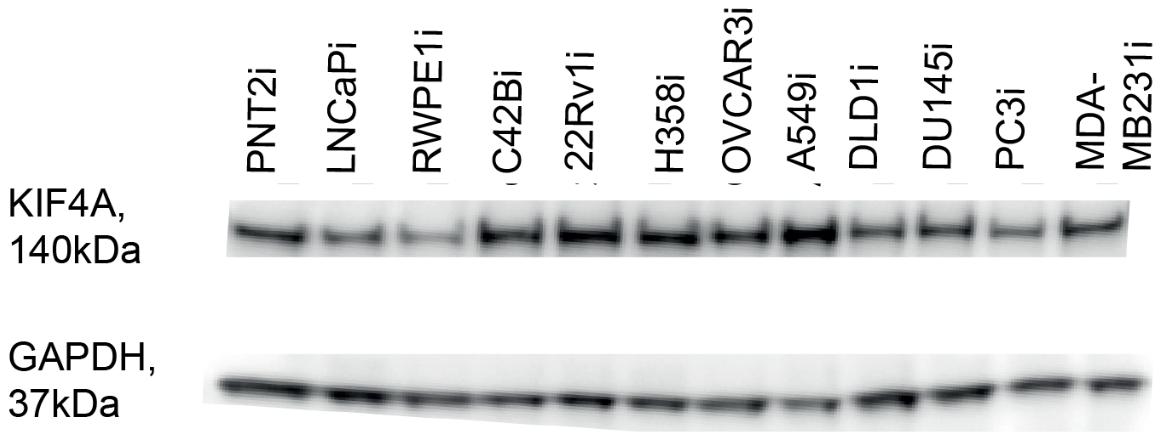

Supplementary Figure 14: Transcriptome analysis of LNCaPi cells following *KIF4A* knockdown and control. (A) and (B) KIF4A knockdown efficiency at mRNA and protein level, respectively. mRNA expression was measured by qPCR was performed (n=3 as biological replicates; Mean± SEM; Unpaired two-tailed t-test was used to determine statistical significance. (C) Volcano plot showing statistical (p-value) and magnitude of change (fold change). Benjamini-Hochberg corrected p-value < 0.05 and log2 foldchange > 0.5 or < 0.5 were considered statistically significant; (D) Ranking of Hallmark gene signatures enriched following *KIF4A* knockdown; (E) Gene Set Enrichment Analysis of *MYC* and *E2F* signatures with *KIF4A*.

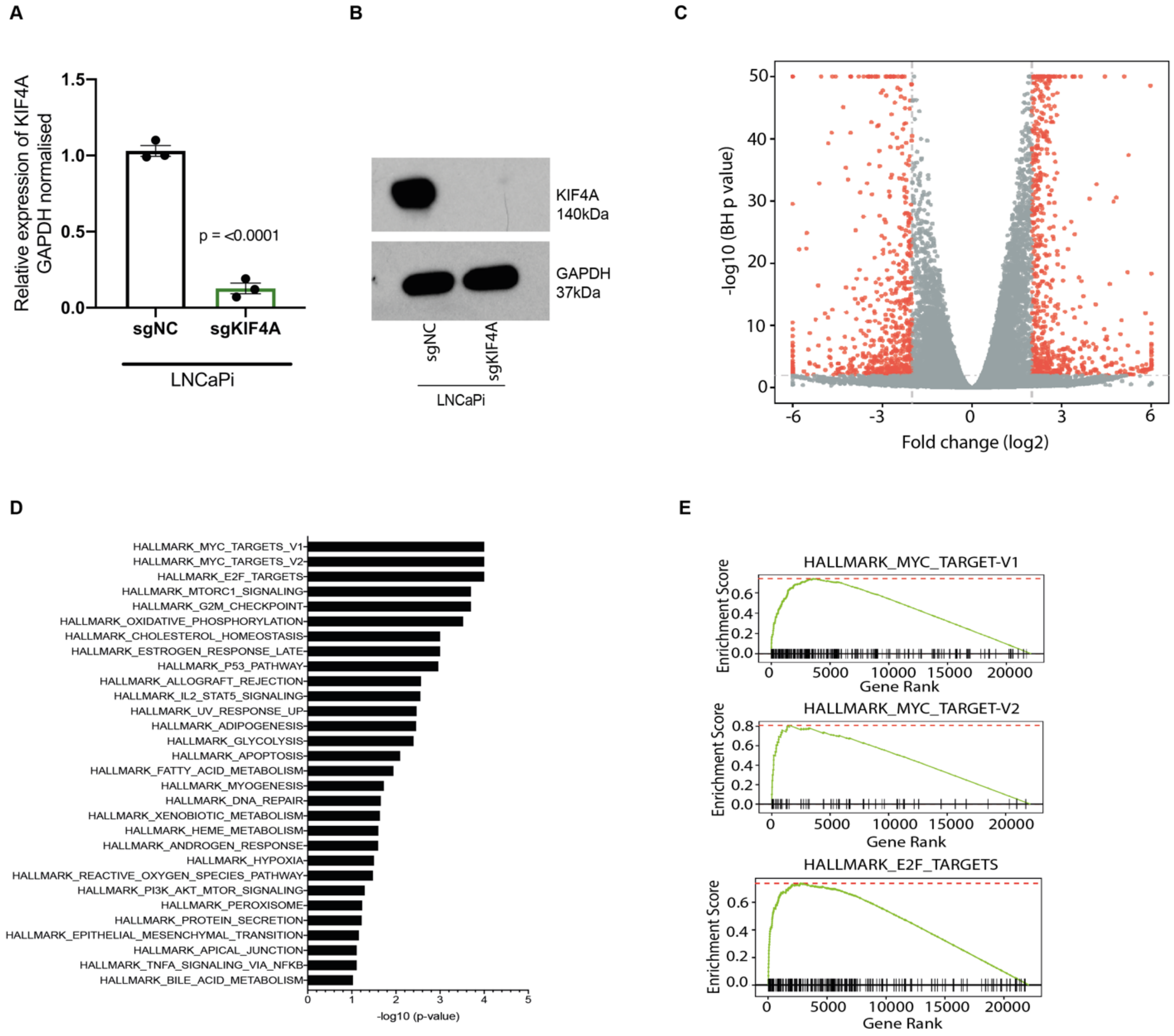

Supplementary Figure 15: Quality control of ATAC-Sequencing performed in LNCaPi and C42Bi cells with *KIF4A* knockdown and control. (A) Correlation between replicates; (B) Principal component analysis (PCA) analysis plot; (C) Peaks annotation in each sample, (D) and (E) Visualization of *AR* and *MYC* peaks in the samples sequenced as examples.

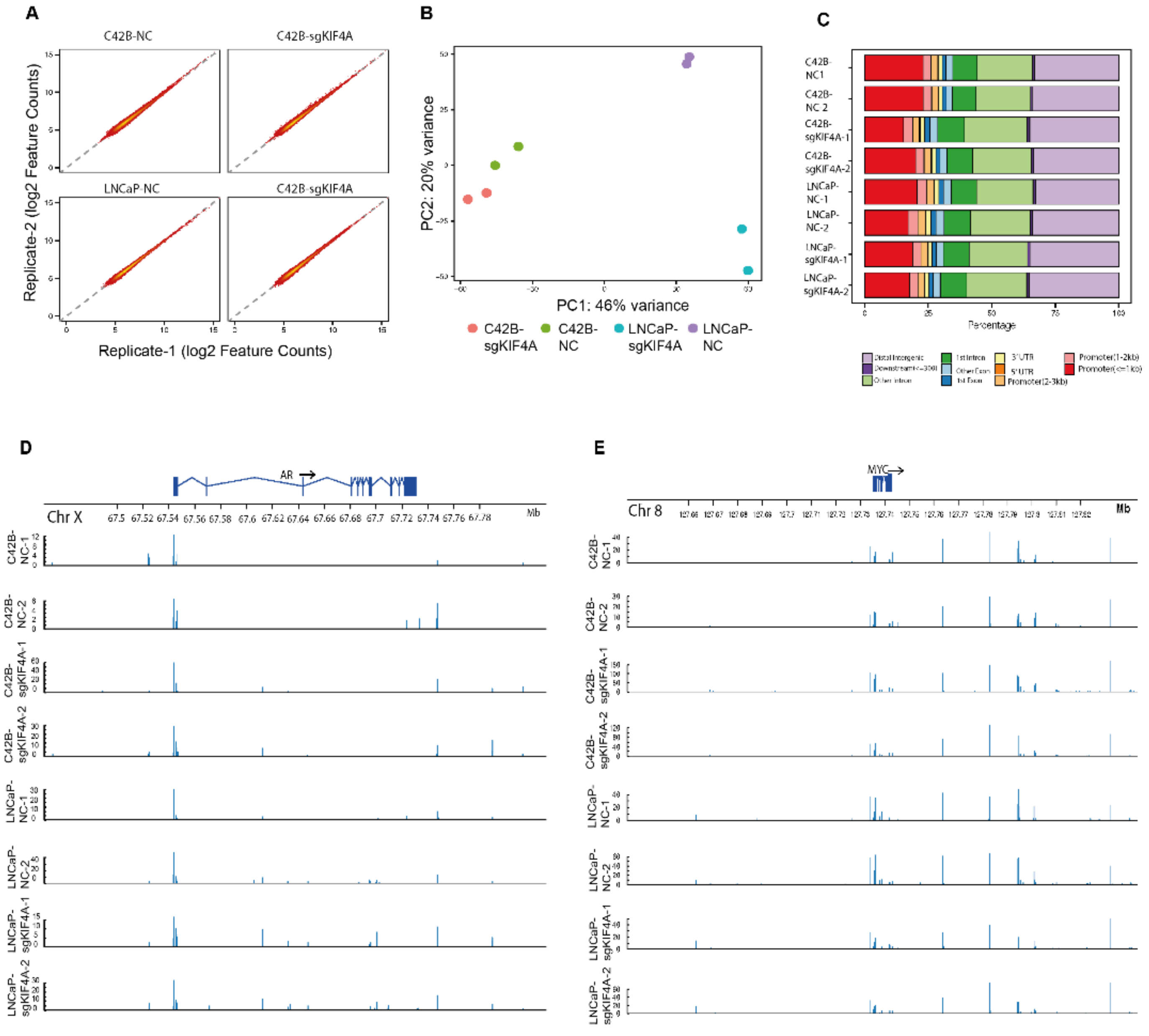

Supplementary Figure 16: ATAC-Sequencing peaks enrichment. (A) and (C)Volcano plot showing gain and loss of open chromatin following knockdown of *KIF4A* in LNCaPi and C42Bi cells, respectively. Benjamini-Hochberg tests were performed for statistical analysis; (B) and (D) Peaks enrichment with Hallmark gene signatures following knockdown of *KIF4A* in LNCaPi and C42Bi cells, respectively.

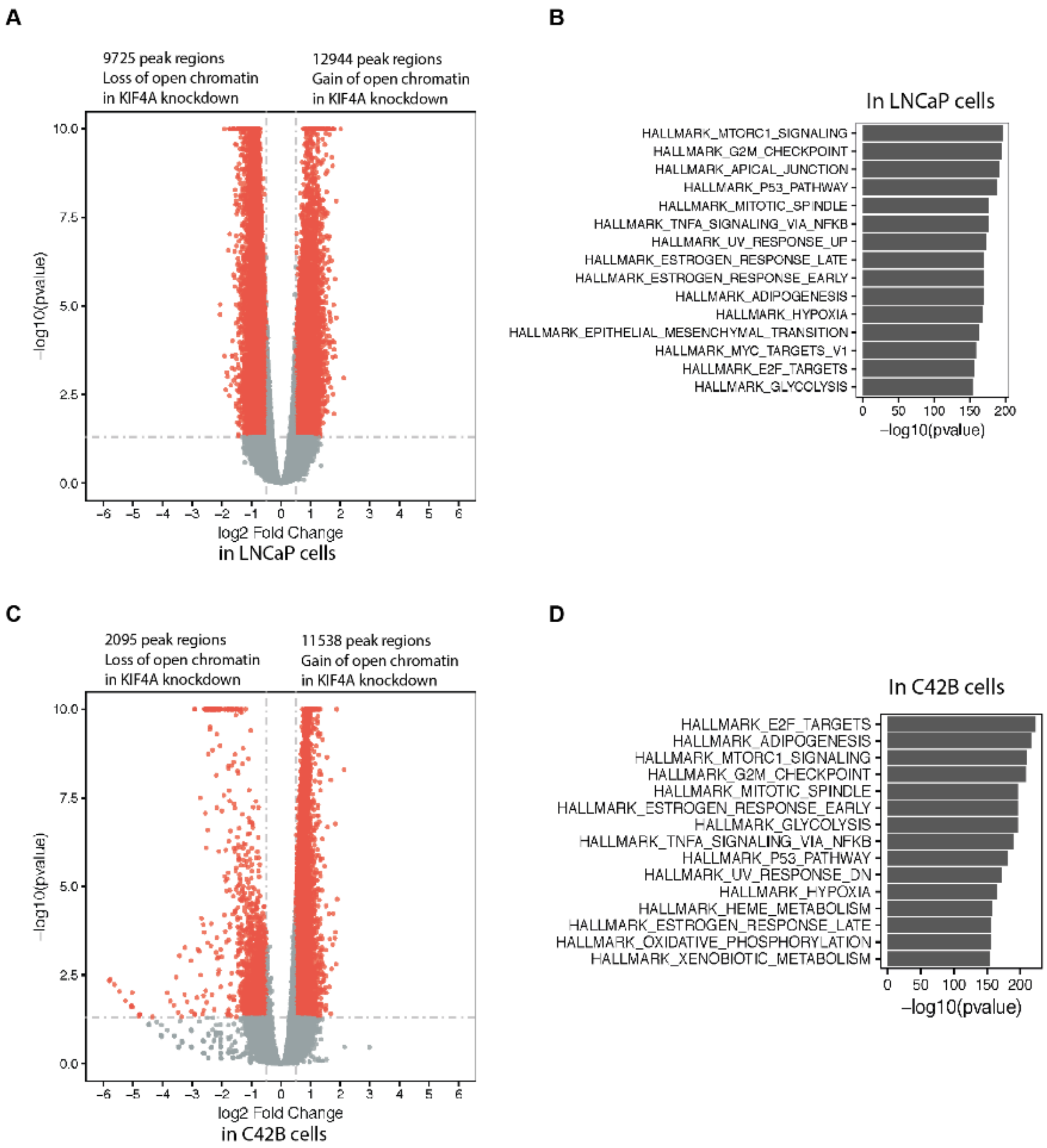

Supplementary Figure 17: Expression of WDR62 in (A) the prostate cancer TCGA cohort with benign tissue (N = 43) and primary prostate cancer (N = 333) and (B) the MSKCC cohort with benign tissue (N = 29), primary prostate cancer (N = 131) and metastatic prostate cancer (N = 19). Two-sided pairwise Wilcoxon rank-sum tests were performed without adjustment for multiple comparisons to test for statistical significance. Boxplot shows median with hinges at 25th and 75th percentiles and whiskers at largest/smallest value within 1.5 * inter quartile range. Individual data points are plotted as points together with the "violin" as a mirrored density plot.

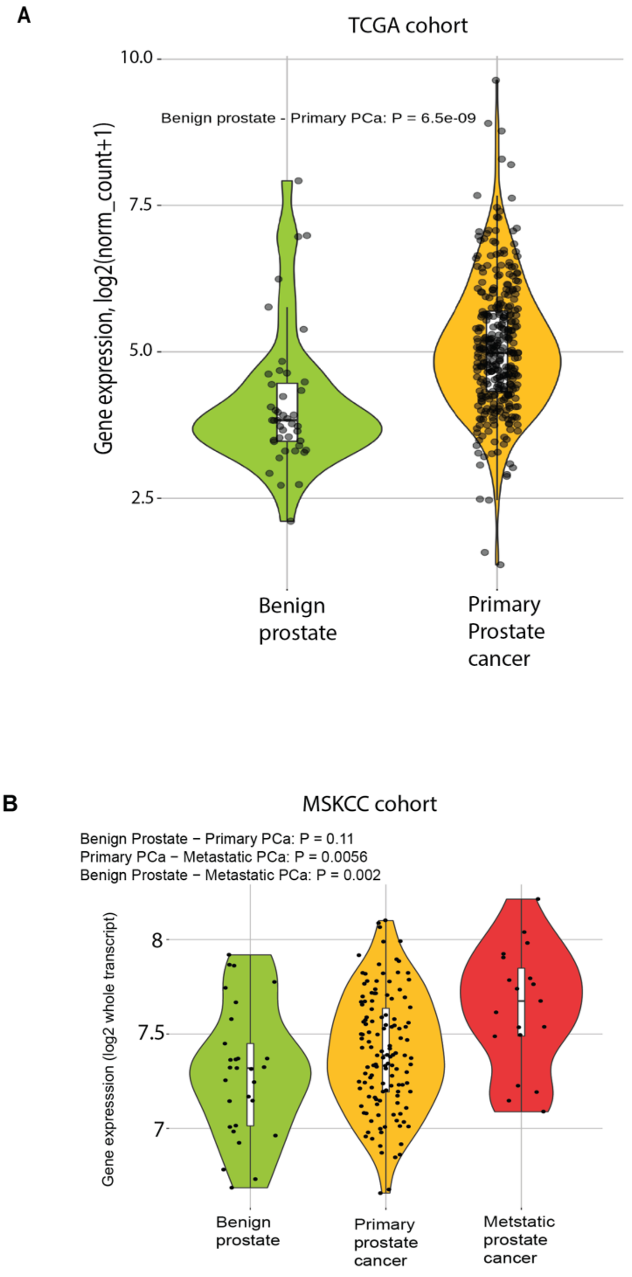

Supplementary Figure 18: Basal expression of WDR62 protein in five prostate cancer cell line models (A). Western blot experiment was performed twice to determine reproducibility. A graph showing (B) *WDR62* knockdown efficiency in CRISPRi prostate cancer cell-line models and (C) A graph *WDR62* cDNA overexpression efficiency in malignant and benign prostate cell line models were measured by qPCR (n=3 as biological replicates; Mean ± SEM; Unpaired two-tailed t-test was used to determine statistical significance.

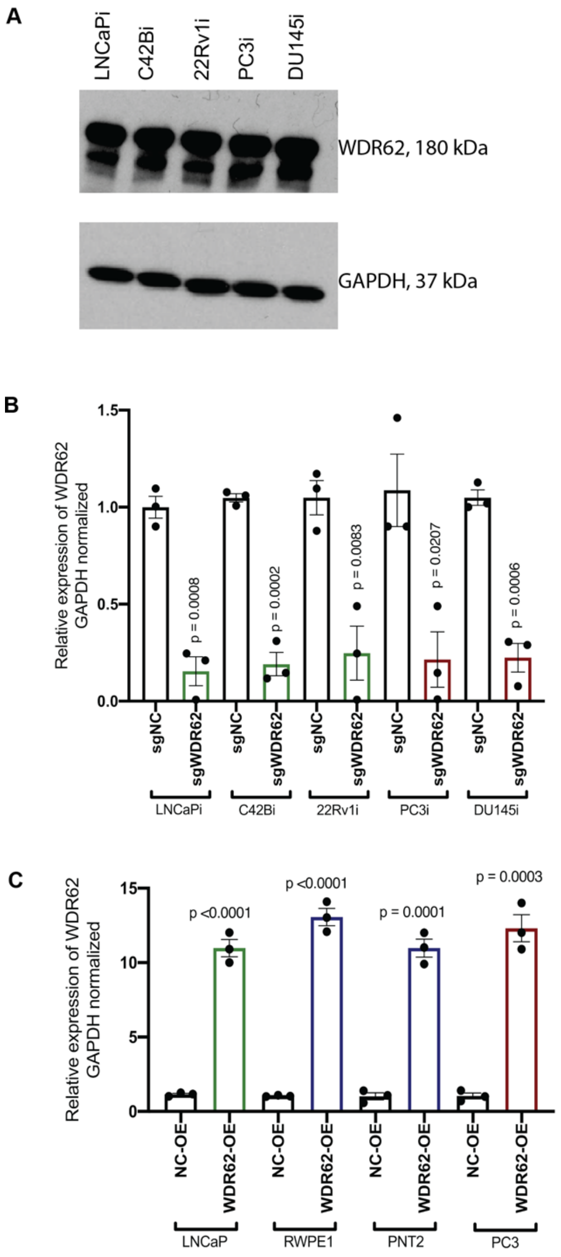

Supplementary Figure 19: Knockdown efficiency of WDR62 in LNCaPi-Dox cells (A) *In vitro* assessment of *WDR62* knockdown relative to control measured by qPCR before the subcutaneous implantation of the cancer cells. Unpaired two-tailed t-test was used to determine statistical significance (n=3 as biological replicates; Mean± SEM; Unpaired two-tailed t-test was used to determine statistical significance). (B) Knockdown efficiency of WDR62 relative to control measured at protein level extracted from the tumors from 4 different mice after 30 days post-implantation. Western blot experiment was performed twice to determine reproducibility.

A

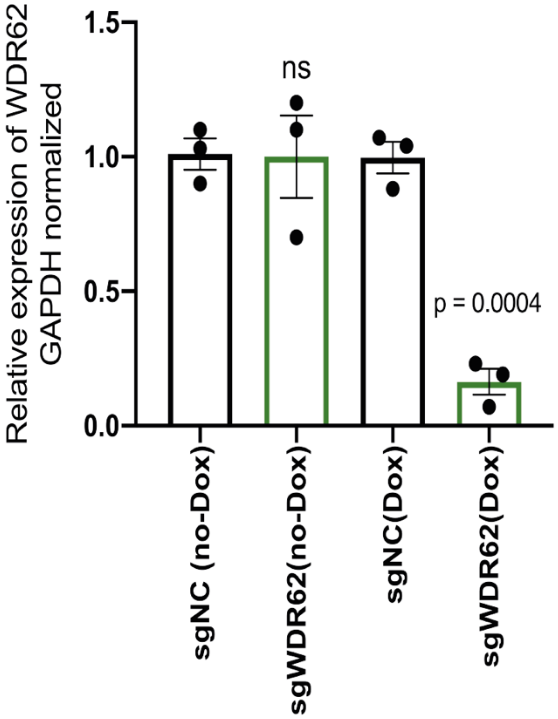

B

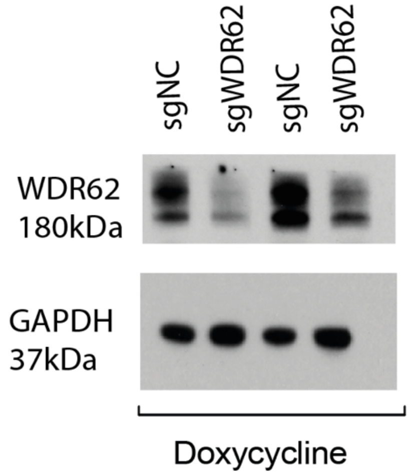

Supplementary Figure 20: Flow cytometry analysis of the effect of *WDR62* knockdown relative to controls on cell cycle in (A) A549i, (B) DLD1i and (C) H358i cell lines. The sgNC control data for cell cycle analyses in Figure 20 A-C are same that of Supplementary Figure 12E-G. (D) A table showing the raw values of the cell cycle assays performed in A549i, DLD1i and H358i cells with WDR62 knockdown and control (n = 2 biological replicates for each cell-line model). (E) A graph showing *WDR62* knockdown efficiency in CRISPRi non-prostate cancer cell line models (n=3 as biological replicates; Mean$\pm$ SEM; Unpaired two-tailed t-test was used to determine statistical significance). (F) Western blot showing basal protein expression of WDR62 in five non-prostate cancer cell line models. Western blot experiment was performed twice to determine reproducibility.

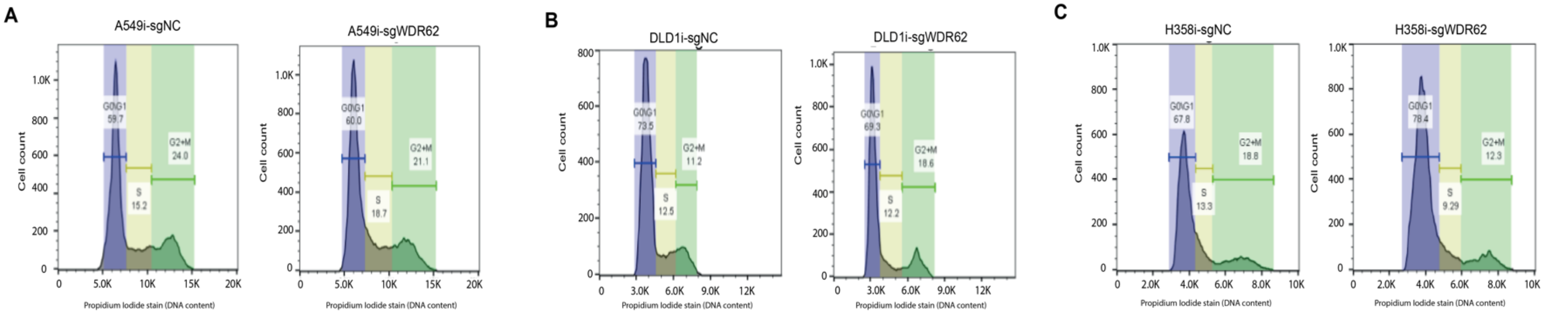

D

Raw values	A549i -sgNC						A549i –sgWDR62					
	G0\G1 (%)	SEM	S (%)	SEM	G2+M (%)	SEM	G0\G1 (%)	SEM	S (%)	SEM	G2+M (%)	SEM
Rep # 1	59.7	4.2	15.2	1.1	24.0	1.7	60.0	6.15	18.7	3.6	21.1	2.3
Rep # 2	68.1		17.4		27.4		72.3		11.5		16.5	
	DLD1i-sgNC						DLD1i-sgWDR62					
	G0\G1 (%)	SEM	S (%)	SEM	G2+M (%)	SEM	G0\G1 (%)	SEM	S (%)	SEM	G2+M (%)	SEM
Rep # 1	73.5	11.3	12.5	1.45	11.2	2.45	69.3	0.95	12.2	0	18.6	0.1
Rep # 2	96.1		15.4		16.1		71.2		12.2		18.4	
	H358i -sgNC						H358i –sgWDR62					
	G0\G1 (%)	SEM	S (%)	SEM	G2+M (%)	SEM	G0\G1 (%)	SEM	S (%)	SEM	G2+M (%)	SEM
Rep # 1	67.8	4.1	13.3	1.4	18.8	2.55	78.4	4.8	9.29	0.55	12.3	0.75
Rep # 2	76.0		10.5		13.7		88.0		10.4		13.8	

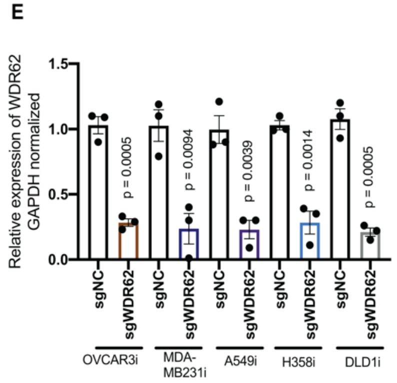

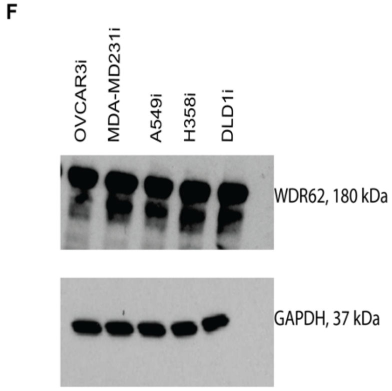

Supplementary Figure 21: Scatter plots showing correlation between (A) *TPX2* and *WDR62* and (B) *AURKA* and *WDR62* in Abida, et al. study. Spearman's and Pearson correlation were performed for statistical analysis. Histograms of pan-cancer essentiality score of (C) AUKRA and (D) TPX2 in DepMap database. The red line denotes the median gene effect of all common essential genes.

A

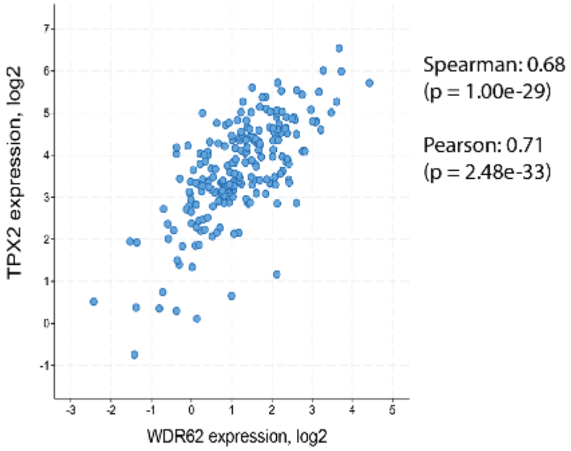

B

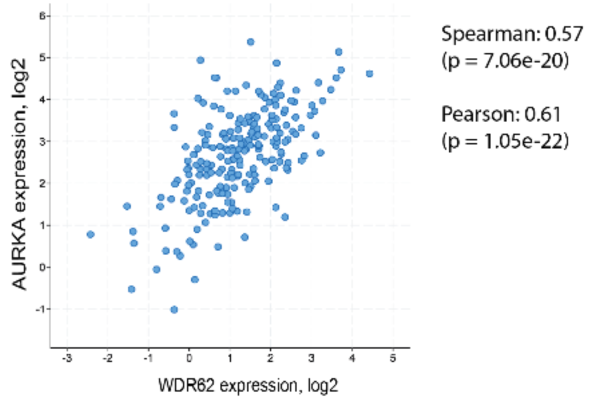

C

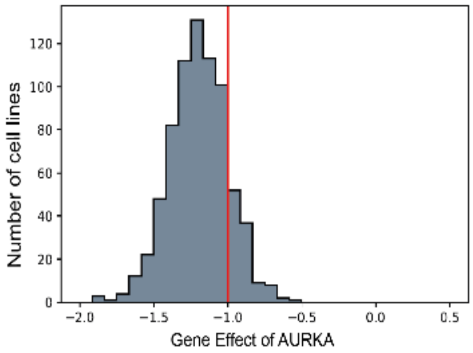

D

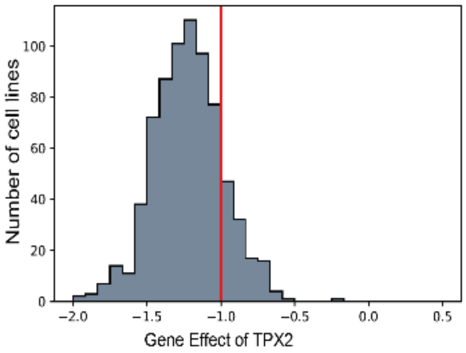

Supplementary Figure 22: Images of uncropped western blots shown in the Figures 4C, 4D and 4E

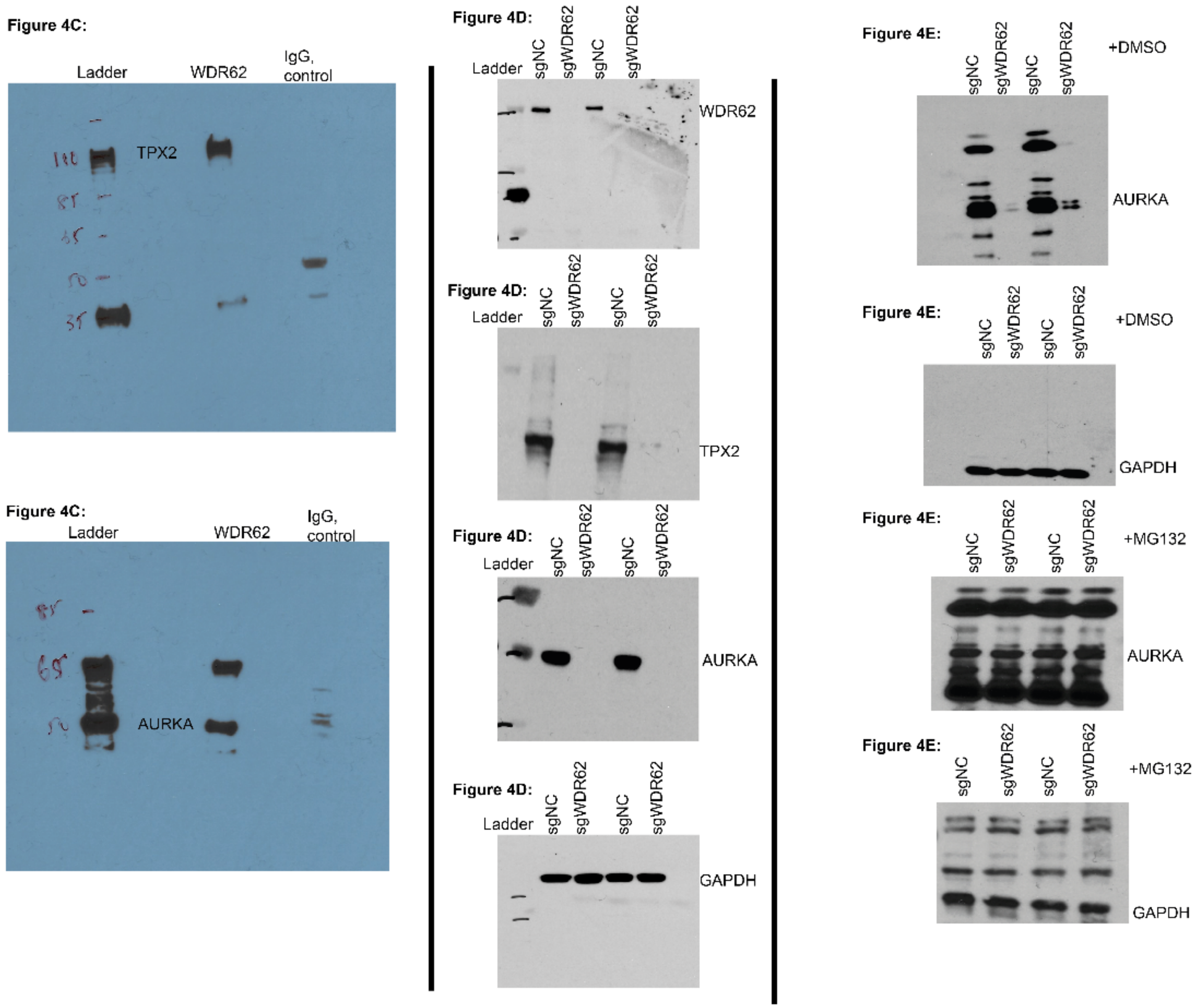

Supplementary Figure 23: Images of uncropped western blots shown in the Supplementary Figures 7B, 8C, 11B, 13, 14B, 18A, 19B and 20F.

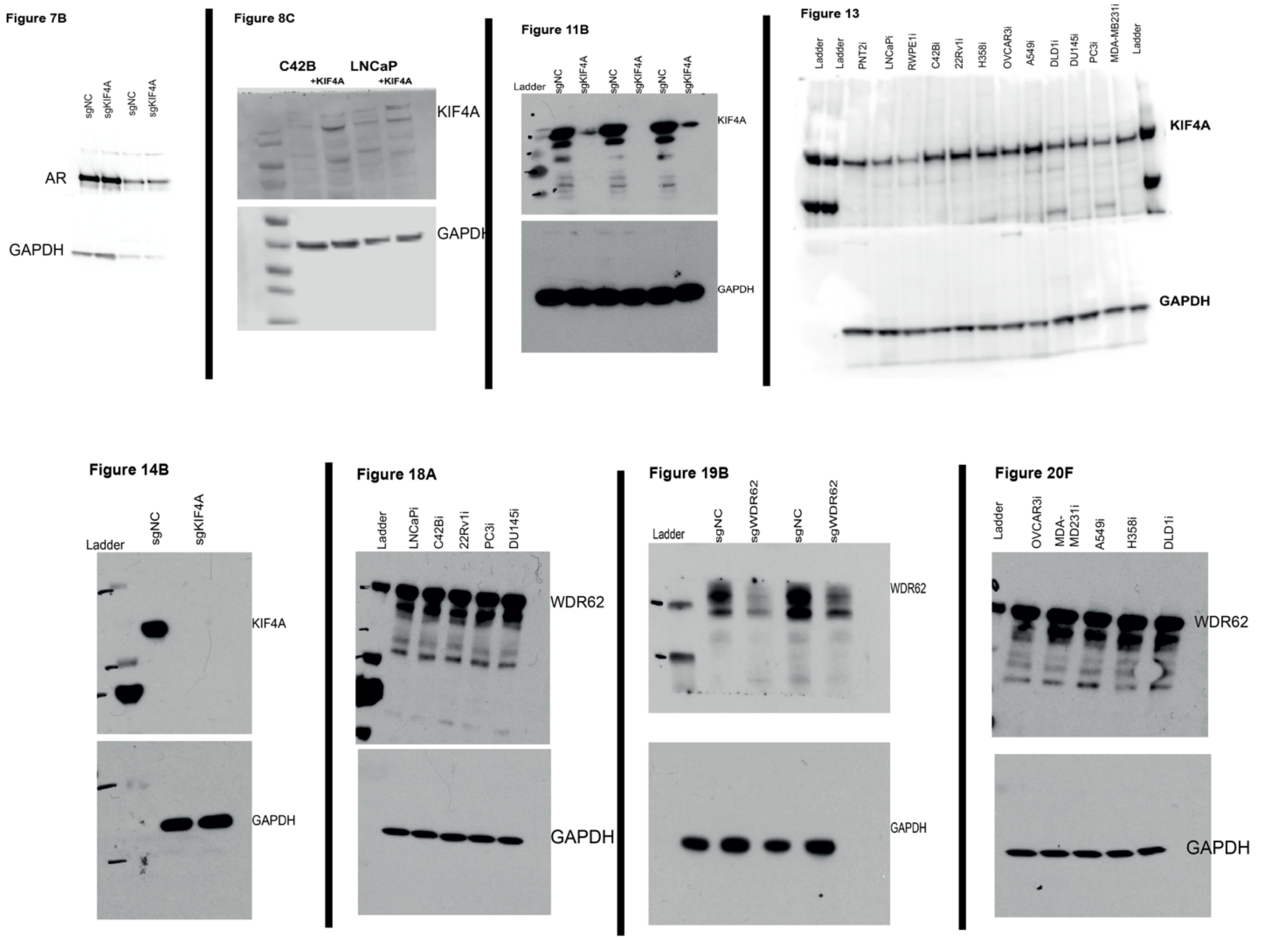

Supplementary Table 1: List of all primers used in the study

Gene name	Forward	Reverse
KIF4A	5′-TGCGTGGTCAAGTTTCGGAGTC-3′	5′- GCTGTAGGTCAGCAATCTGAGC-3′
WDR62	5′- CACTTGCTGGAGATTGACCACC -3′	5′- TCTGGCTCACACTCTTCCTCCA -3′
DPH2	5′- ACCTGGACGGAGTGTACGAG-3′	5′- TCTCCCAATAGCTGGTCAGG-3′
ST3	5′- GATCACGCTCAAGTCCATGG-3′	5′- CTTGCCCAGGTCAGAAGGA-3′
AR	5′- ATGGTGAGCAGAGTGCCCTATC-3′	5′- ATGGTCCCTGGCAGTCTCCAAA -3′
GAPDH	5′- GTCTCCTCTGACTTCAACAGCG -3′	5′- ACCACCCTGTTGCTGTAGCCAA -3′

Supplementary Table 2: List of all sgRNAs used in the study

sgRNA name	Sequence
KIF4A-1	GTACGCACCTGAGGCTCTCC
KIF4A-2	GGGCCCAGGGAGAACGGGGA
WDR62-1	GCGGCGGTTAGGGGATGTAA
WDR62-2	GGCCTCCGGCGTGACGATGG
AR-1	GCTCCCCGGGATCTCGGAGG
AR-2	GGCTTGCTGGGAGAGCGGGA
NDUFB11-1	GGACCACCATCCACCTACTA
NDUFB11-2	GAGCGCGCTTCCTTAGTAGG
TSR2-1	GGACTGGGGCCGGATAATGG
TSR2-2	GTAGGCGGCATAAGAGGAGT
HTRA2-1	GCGCCCGCCCTACTCAGAGG
HTRA2-2	GGTGCCGCCTCTGAGTAGGG
ICT1-1	GTCGCAAGACCTGAGCATGG
ICT1-2	GCCTGAGCCGAGCCGGAGTC
HOXB13-1	GACCCTGGCCGCGTACCCCG
HOXB13-2	GGGATTCCCCCGGCCTGGGT
ST3-1	GTCACTCCAAGGGAACGGAG
ST3-2	GCCCTGCGCCCGGACTGAAG
DPH2-1	GGGCTGAAGGGGATACTCAC
DPH2-2	GATGTTTAGCAGCCCTGCCG
NC	GAACGACTAGTTAGGCGTGTA
