# Supplementary material for: An integrated functional and clinical genomics approach reveals genes driving aggressive metastatic prostate cancer
Source: Nat Commun. 2021 Jul 29;12:4601. doi: 10.1038/s41467-021-24919-7 (PMC8322386; doi:10.1038/s41467-021-24919-7)
Supplement: Supplementary file 2 — Description of Additional Supplementary Files [file 41467_2021_24919_MOESM2_ESM.pdf]

Description of Additional Supplementary Files

File Name: Supplementary Data 1
Description: Genome scale CRISPRi screen data for LNCaPi and C42Bi cells

File Name: Supplementary Data 2
Description: List of genes (n=1472) that are required for cell proliferation or survival revealed following analysis of LNCaPi screen data.

File Name: Supplementary Data 3
Description: Gene hits (n=36) listed after implementing clinical genomics filters

File Name: Supplementary Data 4
Description: Gene hits listed after implementing functional genomics and clinical data filters: Sheet 1: Genes filtered against PICKLES database; Sheet 2: Narrowed down gene list from sheet 1 filtered against DepMap database; Sheet 3: Narrowed down gene list from sheet 1 and 2 filtered against published two non-prostate cancer cell lines CRISPRi database; Sheet 4: Narrowed down gene list from sheet 1, 2 and 3 filtered against prostate cancer metastasis data (MSKCC cohort (48)).
