# Supplementary material for: An integrated functional and clinical genomics approach reveals genes driving aggressive metastatic prostate cancer
Source: Nat Commun. 2021 Jul 29;12:4601. doi: 10.1038/s41467-021-24919-7 (PMC8322386; doi:10.1038/s41467-021-24919-7)
Supplement: Supplementary file 7 — Reporting Summary [file 41467_2021_24919_MOESM7_ESM.pdf]

nature research

Corresponding author(s): Luke Gilbert and Felix Feng

Last updated by author(s): 6/14/2021

Reporting Summary

Nature Research wishes to improve the reproducibility of the work that we publish. This form provides structure for consistency and transparency in reporting. For further information on Nature Research policies, see our Editorial Policies and the Editorial Policy Checklist.

Statistics

For all statistical analyses, confirm that the following items are present in the figure legend, table legend, main text, or Methods section.

n/a	Confirmed	
☐	☒	The exact sample size (*n*) for each experimental group/condition, given as a discrete number and unit of measurement
☐	☒	A statement on whether measurements were taken from distinct samples or whether the same sample was measured repeatedly
☐	☒	The statistical test(s) used AND whether they are one- or two-sided<br>*Only common tests should be described solely by name; describe more complex techniques in the Methods section.*
☐	☒	A description of all covariates tested
☒	☐	A description of any assumptions or corrections, such as tests of normality and adjustment for multiple comparisons
☐	☒	A full description of the statistical parameters including central tendency (e.g. means) or other basic estimates (e.g. regression coefficient) AND variation (e.g. standard deviation) or associated estimates of uncertainty (e.g. confidence intervals)
☐	☒	For null hypothesis testing, the test statistic (e.g. *F*, *t*, *r*) with confidence intervals, effect sizes, degrees of freedom and *P* value noted<br>*Give P values as exact values whenever suitable.*
☒	☐	For Bayesian analysis, information on the choice of priors and Markov chain Monte Carlo settings
☒	☐	For hierarchical and complex designs, identification of the appropriate level for tests and full reporting of outcomes
☐	☒	Estimates of effect sizes (e.g. Cohen's *d*, Pearson's *r*), indicating how they were calculated

Our web collection on statistics for biologists contains articles on many of the points above.

Software and code

Policy information about availability of computer code

Data collection	Not applicable
Data analysis	A description of all custom code used for analysis is provided in the methods. Custom code for integrating bespoke CRISPR screens with DepMap data is provided at https://github.com/GilbertLabUCSF/CanDI. Additional custom code will be provided upon request. Published code used for analysis is cited appropriately. ImageJ Bundle with 64 bit with Java 1.8.0_172 was used for colony formation and anchorage independent growth assay analysis. Attune NxT flow cytometry software V3.1 was used for all flow cytometry analysis. Incucyte Base Software provided by the Incucyte system of EssenBioscience was used to analyze the scratch wound assay.

For manuscripts utilizing custom algorithms or software that are central to the research but not yet described in published literature, software must be made available to editors and reviewers. We strongly encourage code deposition in a community repository (e.g. GitHub). See the Nature Research guidelines for submitting code & software for further information.

Data

Policy information about availability of data

All manuscripts must include a data availability statement. This statement should provide the following information, where applicable:

- Accession codes, unique identifiers, or web links for publicly available datasets
- A list of figures that have associated raw data
- A description of any restrictions on data availability

Data Availability: All data generated or analyzed during this study are included in this published article (and its supplementary information files) or have been publicly deposited as indicated. PICKLES (http://pickles.hart-lab.org) and DepMap (https://depmap.org/portal/) data are publicly available.

Code Availability: Custom code used in this manuscript is publicly available at https://github.com/GilbertLabUCSF/CanDI or freely available on request.

Field-specific reporting

Please select the one below that is the best fit for your research. If you are not sure, read the appropriate sections before making your selection.

- [x] Life sciences
- [ ] Behavioural & social sciences
- [ ] Ecological, evolutionary & environmental sciences

For a reference copy of the document with all sections, see nature.com/documents/nr-reporting-summary-flat.pdf

Life sciences study design

All studies must disclose on these points even when the disclosure is negative.

Sample size	No sample size calculations were performed. All samples sizes were chosen according to the accepted standard in the field. Data displayed is 2-3 independent biological experiments with 2-3 technical replicates per biological experiment except as noted. For clinical sample analysis we used all available samples.
Data exclusions	No data related to experiments in the manuscript were excluded.
Replication	We used 2-3 biological replicates per experiment generally with as 2-3 technical replicates per experiment. Exact number of samples was previously provided to the Nature Communications and senior editor Dr. Sundaram. All attempts at replication were successful.
Randomization	Randomization is not relevant to the clinical or experimental studies that were performed. Randomization is not standardly performed in this field of biology research.
Blinding	Blinding was not performed however a number of experiments were independently replicated by co-authors to ensure reproducibility. Blinded experiments are not standardly performed in this field of experimental biology research.

Reporting for specific materials, systems and methods

We require information from authors about some types of materials, experimental systems and methods used in many studies. Here, indicate whether each material, system or method listed is relevant to your study. If you are not sure if a list item applies to your research, read the appropriate section before selecting a response.

Materials & experimental systems

n/a	Involved in the study	
[ ]	[x]	Antibodies
[ ]	[x]	Eukaryotic cell lines
[x]	[ ]	Palaeontology and archaeology
[ ]	[x]	Animals and other organisms
[x]	[ ]	Human research participants
[x]	[ ]	Clinical data
[x]	[ ]	Dual use research of concern

Methods

n/a	Involved in the study	
[x]	[ ]	ChIP-seq
[ ]	[x]	Flow cytometry
[x]	[ ]	MRI-based neuroimaging

Antibodies

Antibodies used	Antibodies used were: KIF4A (Thermo, Cat# PA5-30492), WDR62 (Bethyl Labs, Cat#A301-560A), GAPDH (14C10) (Cell signaling, Cat#2118), Aurora A (D3E4Q) (Cell signaling, Cat #14475), TPX2 (Novus Bio, Cat#NB500-179), Androgen Receptor (D6F11) (Cell Signaling #5153)
Validation	Antibody specificity for primary antibodies was validated by CRISPRi knockdown experiments. All antibodies were raised for human proteins and used to detect human proteins.

Eukaryotic cell lines

Policy information about cell lines

Cell line source(s)	LNCaP, C42B, 22Rv1, PC3, DU145, RWPE1, OVCAR3, MDA-MB-231, A549, H358, DLD1 cell lines were purchased from ATCC. PNT2 cells were purchased from Millipore Sigma
Authentication	Cell lines were authenticated by STR analysis yearly.
Mycoplasma contamination	All cell lines tested negative for mycoplasma. Mycoplasma testing was performed every 3-6 months.

Commonly misidentified lines (See ICLAC register)	No commonly misidentified cell lines were used in this study.

Animals and other organisms

Policy information about studies involving animals; ARRIVE guidelines recommended for reporting animal research

Laboratory animals	NSG male mice 8-10 weeks of age were used for all experiments.
Wild animals	No wild animals were used in this study.
Field-collected samples	No field-collected samples were used in this study.
Ethics oversight	UCSF IACUC approval was obtained prior to all experiments.

Note that full information on the approval of the study protocol must also be provided in the manuscript.

Flow Cytometry

Plots

Confirm that:

- [x] The axis labels state the marker and fluorochrome used (e.g. CD4-FITC).
- [x] The axis scales are clearly visible. Include numbers along axes only for bottom left plot of group (a 'group' is an analysis of identical markers).
- [x] All plots are contour plots with outliers or pseudocolor plots.
- [x] A numerical value for number of cells or percentage (with statistics) is provided.

Methodology

Sample preparation	All flow cytometry data is derived from cell lines purchased from ATCC as listed in the Methods. For cell competition assays the cells were trypsinized, trypsin was quenched with PBS + FBS, the cells were washed in PBS + FBS and then analyzed. For cell cycle analysis, the cells were trypsinized, trypsin was quenched with PBS + FBS, the cells were washed in PBS, counted, fixed with 70% ethanol and stained with Propidium Iodide and then analyzed.
Instrument	Life Technologies/Invitrogen Attune NxT flow cytometer
Software	Life Technologies/Invitrogen Attune NxT flow cytometry software v3.1
Cell population abundance	A minimum of 10,000 cells were analyzed for all flow cytometry experiments.
Gating strategy	All samples were gated on FSC/SSC to identify and analyze live cells and exclude dead/dying cells and cell debris. Boundaries for cell cycle analysis are shown in each figure.

- [x] Tick this box to confirm that a figure exemplifying the gating strategy is provided in the Supplementary Information.
